# Supplementary material for: Chemoproteogenomic stratification of the missense variant cysteinome
Source: Nat Commun. 2024 Oct 28;15:9284. doi: 10.1038/s41467-024-53520-x (PMC11519605; doi:10.1038/s41467-024-53520-x)
Supplement: Supplementary file 1 — Supplementary Information [file 41467_2024_53520_MOESM1_ESM.pdf]

Supporting Information

Chemoproteogenomic stratification of the missense variant cysteinome

Heta Desai[1,2], Katrina H. Andrews[1], Kristina V. Bergersen[3], Samuel Ofori[1], Fengchao Yu[4], Flowreen Shikwana[1,5], Mark Arbing[5,6], Lisa M. Boatner[1,5], Miranda Villanueva[1,2], Nicholas Ung[1], Elaine F. Reed[3], Alexey I. Nesvizhskii[4,8], Keriann M. Backus[1,2,5,7,8,9]

AFFILIATIONS:

1. Biological Chemistry Department, David Geffen School of Medicine, UCLA, Los Angeles, CA, 90095, USA.
2. Molecular Biology Institute, UCLA, Los Angeles, CA, 90095, USA.
3. Department of Pathology and Laboratory Medicine, David Geffen School of Medicine, UCLA, Los Angeles, CA, 90095, USA
4. Department of Computational Medicine and Bioinformatics, University of Michigan, Ann Arbor, MI, 48109, USA.
5. Department of Chemistry and Biochemistry, UCLA, Los Angeles, CA, 90095, USA.
6. DOE Institute for Genomics and Proteomics, UCLA, Los Angeles, CA, 90095, USA.UCLA
7. Department of Pathology, University of Michigan, Ann Arbor, MI, 48109, USA.
8. Eli and Edythe Broad Center of Regenerative Medicine and Stem Cell Research, UCLA, Los Angeles, CA, 90095, USA.
9. Jonsson Comprehensive Cancer Center, UCLA, Los Angeles, CA, 90095, USA.

*Corresponding Author: kbackus@mednet.ucla.edu

Contents

(A) Supplementary Discussion - 3

(B) Supplementary Figures - 4

(C) Supplementary Note: Data analysis - 40

(D) Supplementary Note: Synthesis of compounds - 41

(E) Supplementary Note Running 2-stage searches with FragPipe - 53

(F) References - 55

(A) Supplementary Discussion

FragPipe GUI with improved 2-stage search. FragPipe generates a "fragpipe-second-pass.workflow" and a "fragpipe-files-second-pass.fp-manifest" after the first search. The manifest file points to the calibrated mzML files generated from the first pass. The workflow file has mass calibration and optimization turned off. Thus, using those two files, the second-pass search skips the calibration and searches the calibrated data. For more details see https://fragpipe.nesvilab.org/docs/tutorial_two_pass_search.html. In the first stage pass, with the "write sub mzML" option enabled, FragPipe utilizes MSFragger[1,2] for mass calibration, search parameter optimization, and database searching. Following this, FragPipe applies MSBooster[3] to compute the deep-learning score, Percolator[4] for PSM rescoring, ProteinProphet[5] for protein inference, and Philosopher for FDR filtering. Subsequently, FragPipe generates new mzML files, which include the scans that did not pass the FDR filtering (default is 1%) and those with a probability higher than a predefined threshold (default is 0). In the second search, as the mass spectral files have already been calibrated and only scans that remained unidentified in the first search have been retained, the mass calibration should be disabled. Moreover, Percolator modeling might fail in the second pass due to a lack of sufficient number of high-scoring PSMs. Therefore, FragPipe lets Percolator reuse the model from the initial pass. FragPipe then generates a new workflow file containing optimized parameters, and a new manifest file with the new (subset) mzML files specified for the second-pass search. The user is merely required to load these two files without needing any further adjustments.

(B) Supplementary Figures

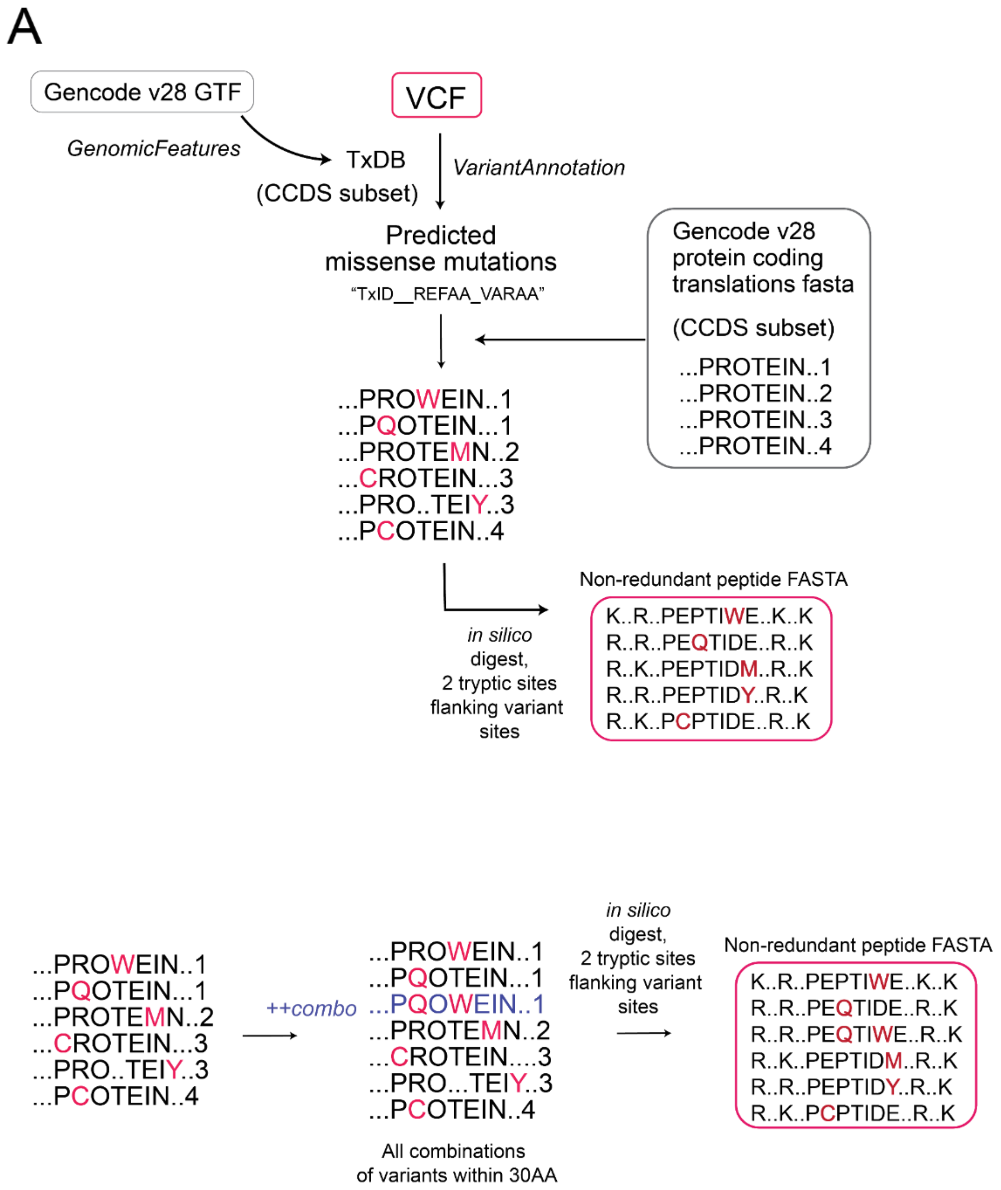

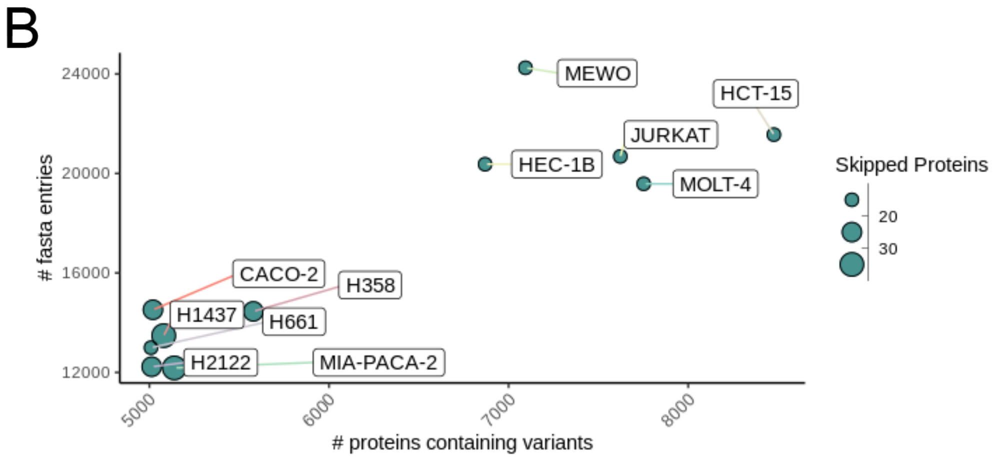

Supplementary Figure 1: Details of database generation. A) Pipeline of packages and inputs to obtain variant peptide databases–*VariantAnnotation*[6] package is used to obtain predicted changes that replace reference sequence residues from Gencode by matching internal transcript IDs (TxID). Details in methods. B) Output from panel A includes combinations of variants with 30AA. Combinations are omitted from proteins with > 25 or 15 variants (top). Variant database sizes calculated as the number of FASTA entries.The skipped proteins indicate the number of proteins that were omitted from combinations in Supplementary Data S4 (tab 30). Source data are provided as a Source Data file.

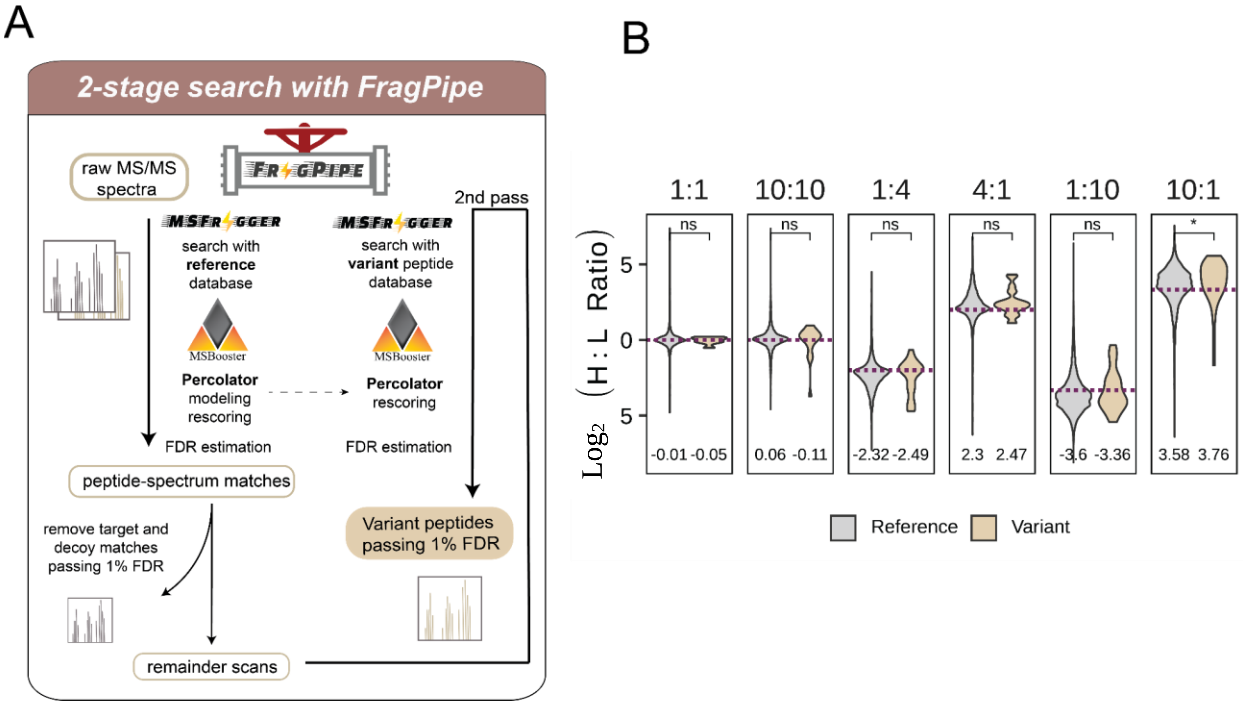

Supplementary Figure 2. 2-stage search implemented into FragPipe GUI with Percolator rescoring A) 2-stage search incorporation into FragPipe GUI workflow. B) Heavy to light ratios (H:L) from triplicate datasets comparing identifications from reference and variant searches; mean ratio value indicated, *dashed lines* indicate ground-truth $\log_2$ ratio, statistical significance was

calculated using Mann-Whitney U test, * $p < 0.05$, ** $p < 0.01$, ns $p > 0.05$. Data provided in Supplementary Data S1.

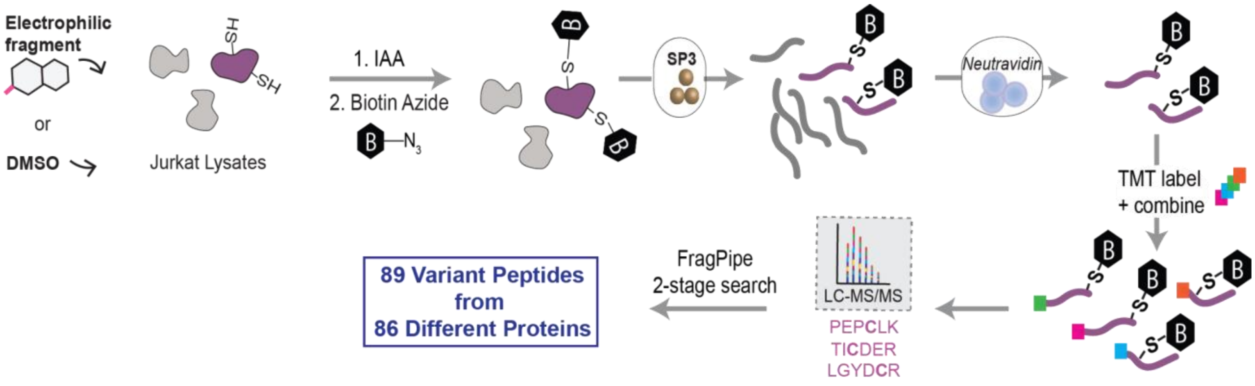

Supplementary Figure 3. Application of TMT tags and subsequent 2-stage search. Jurkat lysates are treated with DMSO or cysteine reactive compounds[7] (KB2, KB3, JC19) in duplicate. Lysates were chased with IAA,clicked with biotin azide, and taken through SP3 sample preparation. Enriched samples were TMT labeled and data was acquired using FAIMS MS2. Samples were searched using our FragPipe 2-stage search to identify 89 variants from 2 runs.

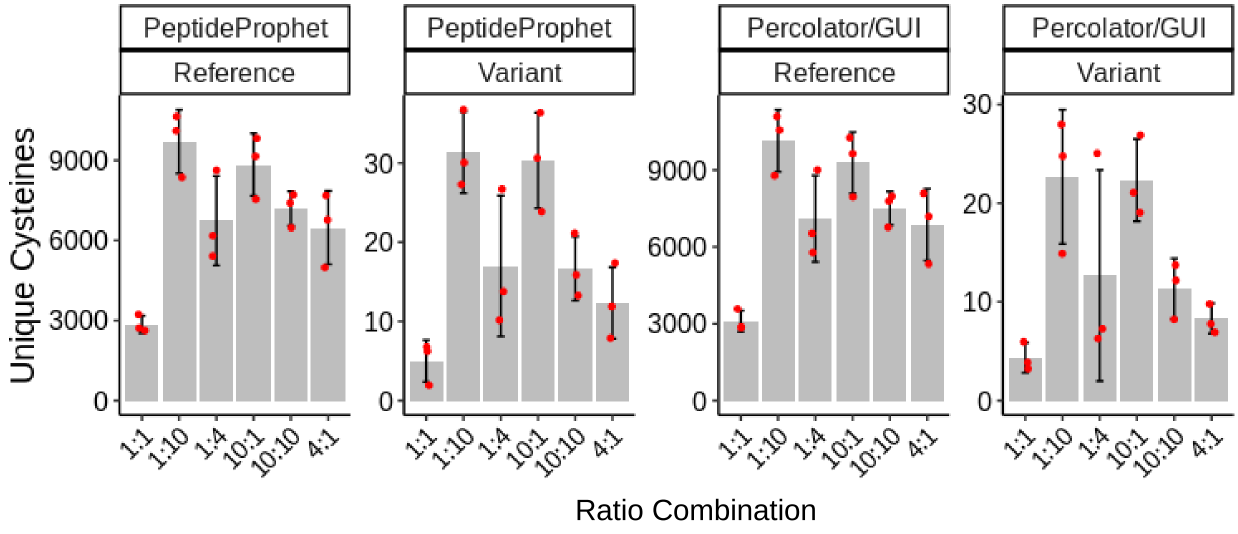

Supplementary Figure 4. Coverage of FragPipe GUI implementing MSBooster with Percolator rescoring in comparison to PeptideProphet command line quantified cysteine identifications from validation datasets. Showing mean values and error bars = SD for triplicate datasets. Source data are provided as a Source Data file.

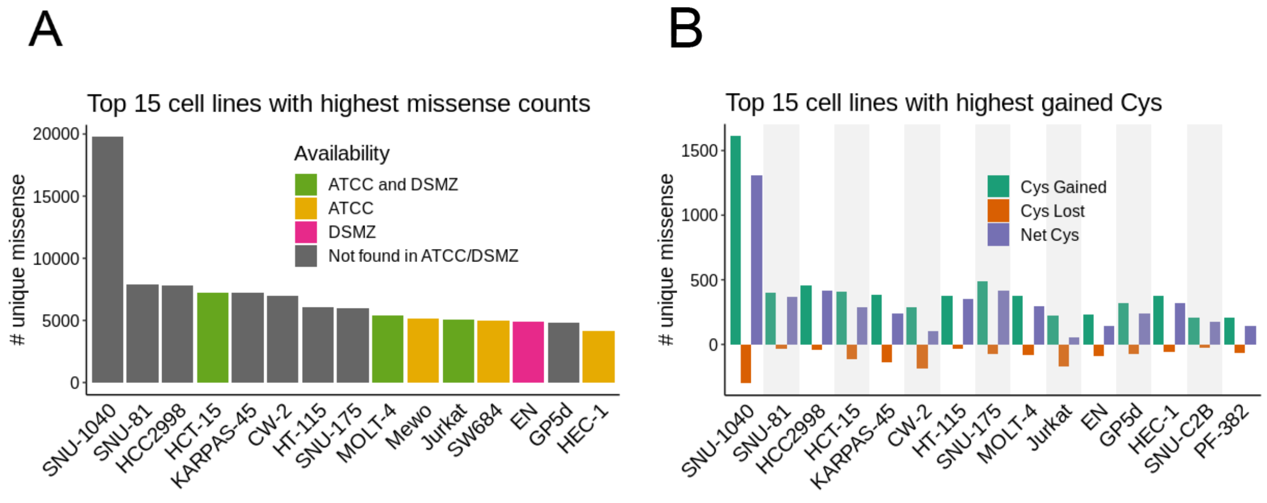

Supplementary Figure 5. Missense counts in Cell Lines found in the Catalogue of Somatic Mutations in Cancer Cell Lines Project database (COSMIC Cell Lines Project release v96) A) The top 15 cell lines with the highest missense burden in COSMIC-CLP. Color indicates availability of high mutational burden cell lines in American Type Culture Collection (ATTC) and German Collection of Microorganisms and Cell Cultures (Deutsche Sammlung von Mikroorganismen und Zellkulturen, DSMZ). B) The top 15 cell lines with the highest gained cysteines showing net gain, total gained and total lost. Source data are provided as a Source Data file.

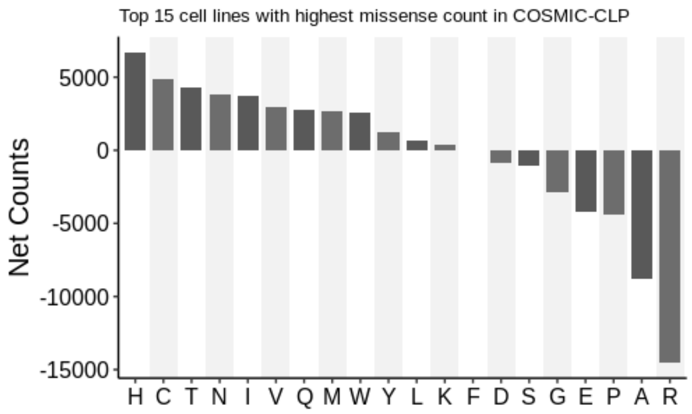

Supplementary Figure 6. Aggregated net amino acid changes (gained counts - lost counts) in the combined top 15 cell lines with highest missense burden in COSMIC-CLP (COSMIC Cell Lines Project release v96). Source data are provided as a Source Data file.

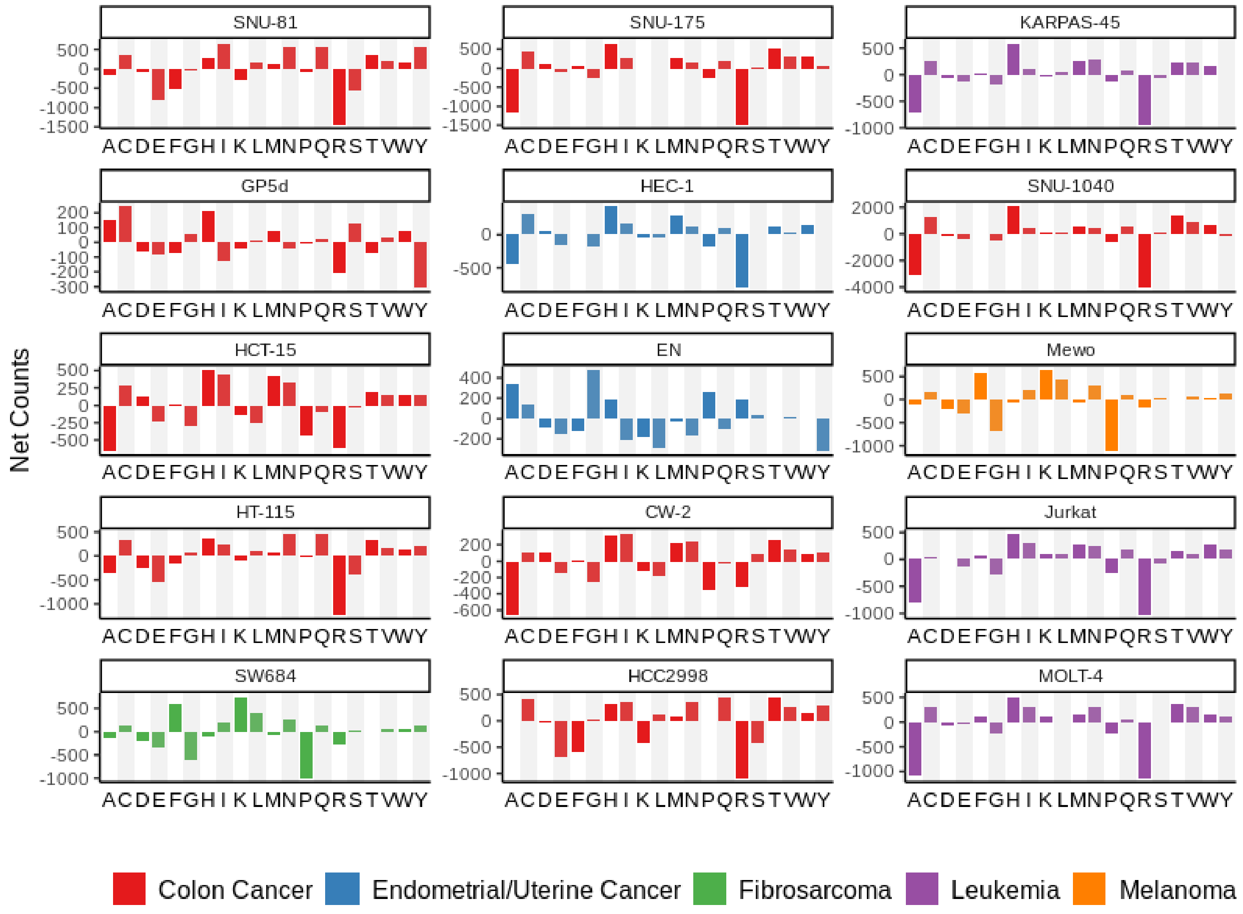

Supplementary Figure 7. Net single amino acid mutation counts (gained counts - lost counts) in top 15 cell lines with highest missense burden (COSMIC Cell Lines Project release v96). Several cell lines show marked depletion of alanine, for example SNU-175, KARPAS-45, Jurkat (A3 subclone) and MOLT-4 cell lines, which represent two colon cancer cell lines, and two T lymphoblast cell lines, respectively. In contrast, several cell lines showed marked net gain of alanine, including EN, Gp5d, and HCC2998 cells, which are an endometrial/uterine cancer line and two colon cancer cell lines, respectively. Source data are provided as a Source Data file.

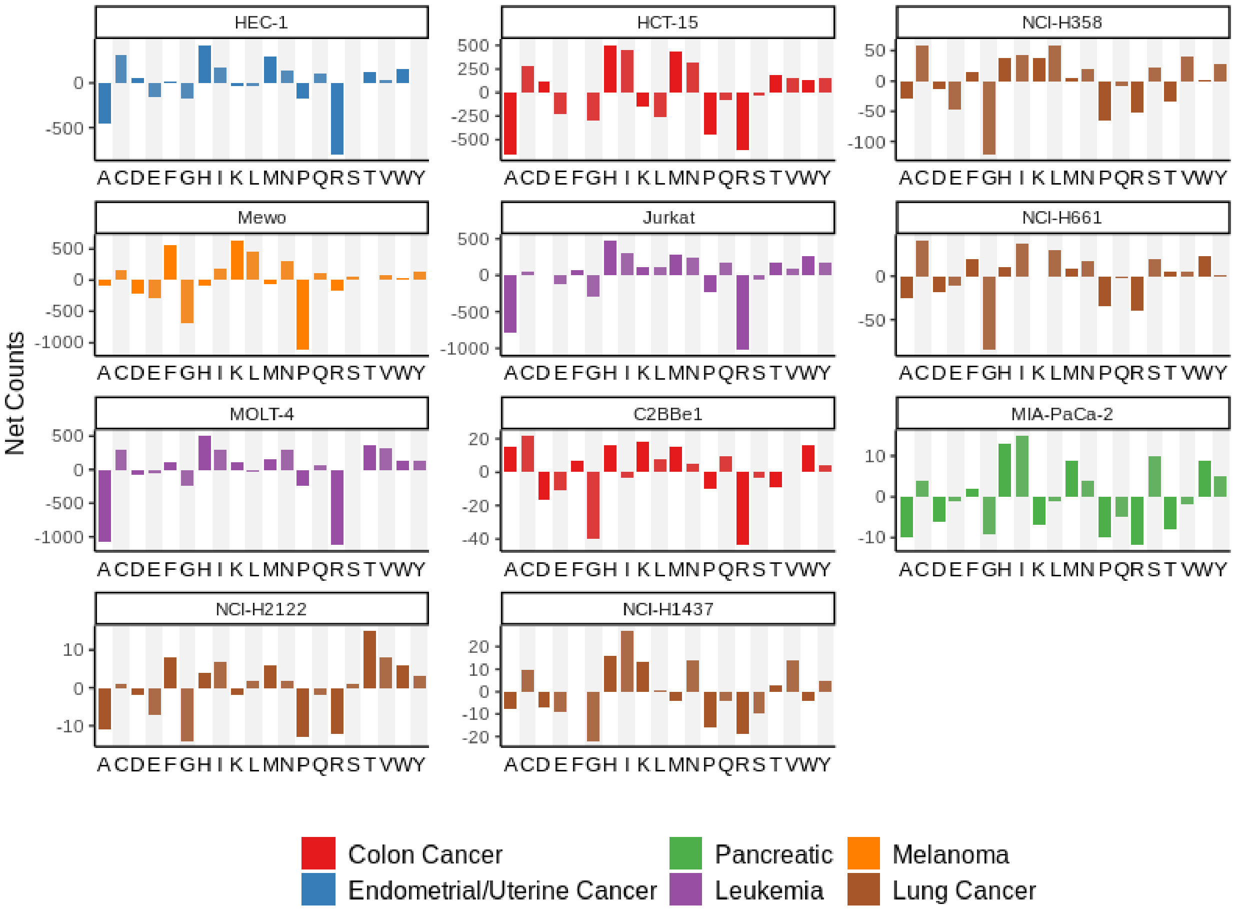

Supplementary Figure 8. Net single amino acid mutation counts (gained counts - lost counts) in the panel of cell lines in this study (COSMIC CLP v96). C2BBe1 is a CaCo-2 cell line clone in COSMIC-CLP. Source data are provided as a Source Data file.

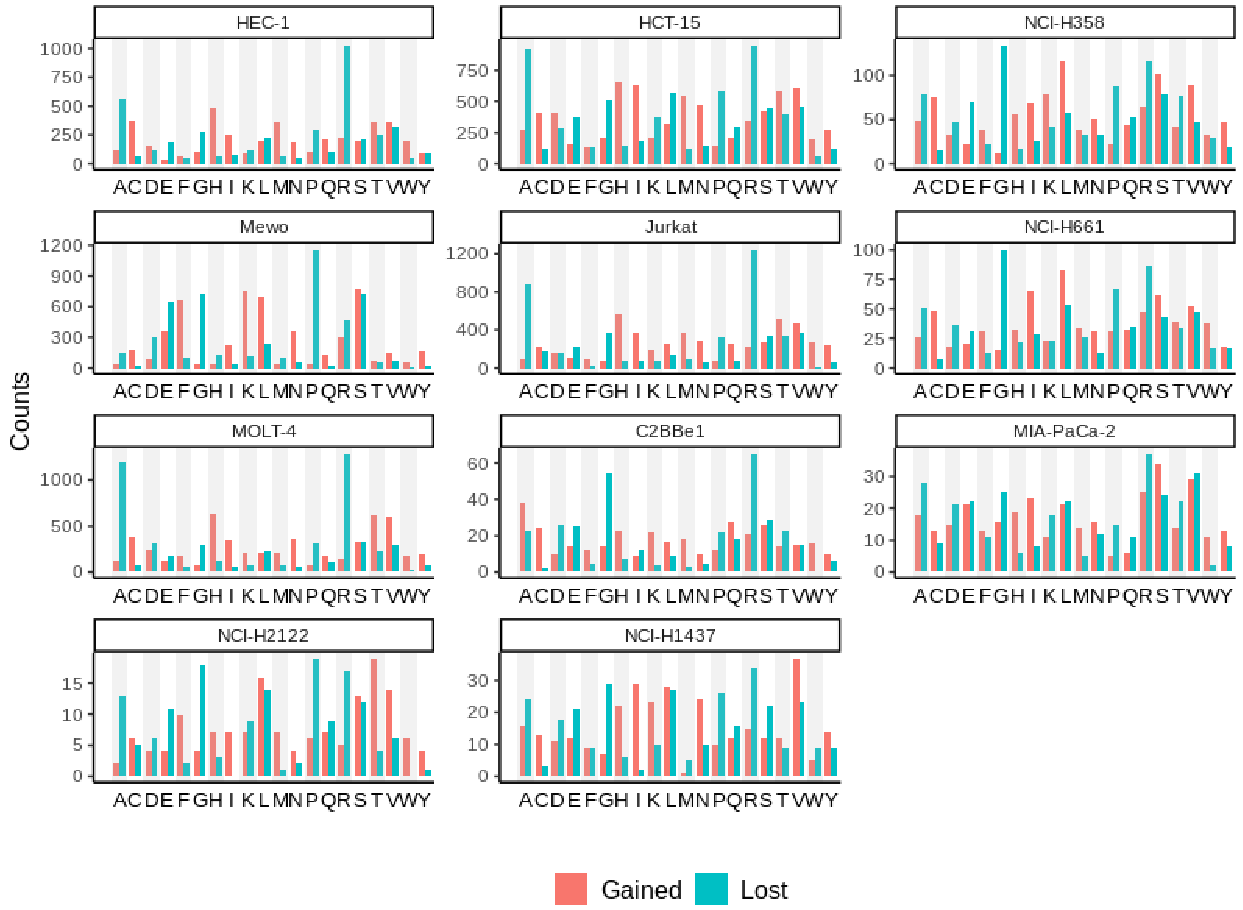

Supplementary Figure 9. Total gain and loss missense mutation counts in a panel of cell lines in this study (COSMIC CLP v96). C2BBe1 is a CaCo-2 cell line clone in COSMIC-CLP. Source data are provided as a Source Data file.

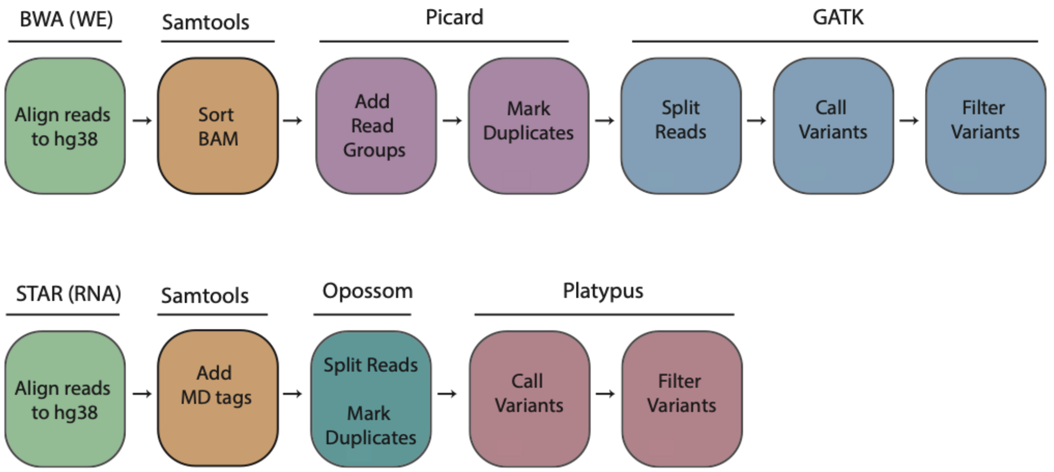

Supplementary Figure 10. Variant calling pipelines for RNA and whole-exome (WE) datasets. Details in methods. Raw reads submitted to Sequence Read Archive (SRA) as BioProject PRJNA997729. Color indicates commands under the toolkit listed.

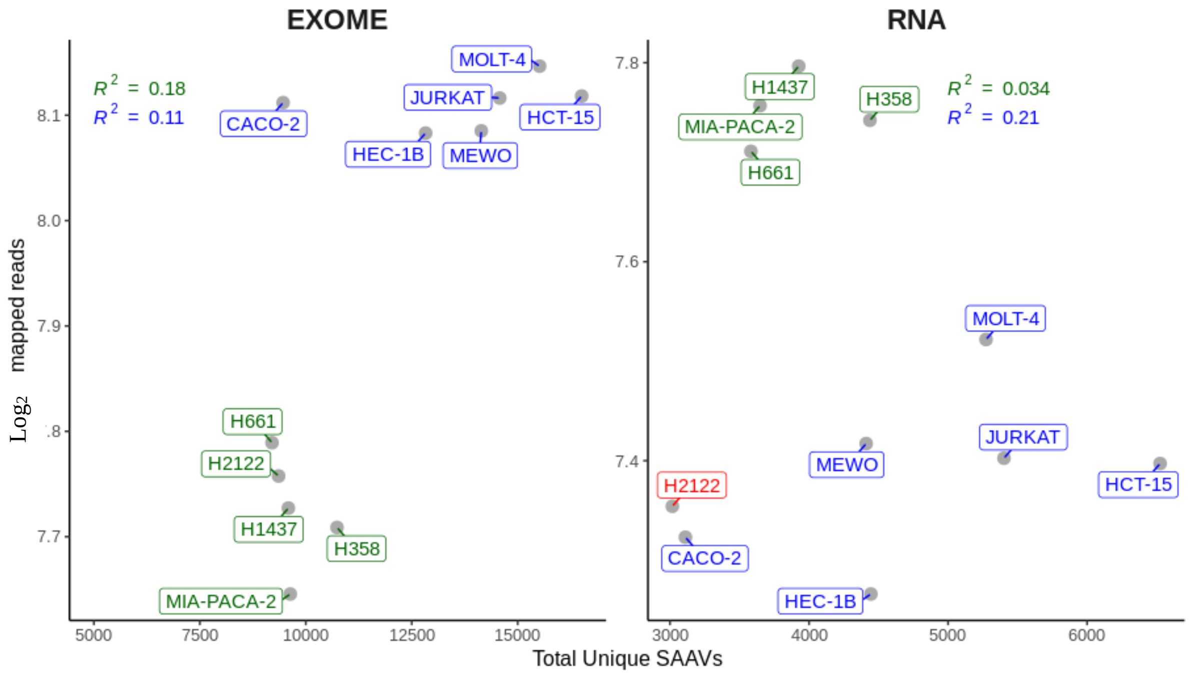

Supplementary Figure 11. Number of mapped reads per cell line after BWA or STAR mapping and total unique SAAVs (single amino acid variants) identified per cell line. Color indicates sequencing batch. Source data are provided as a Source Data file.

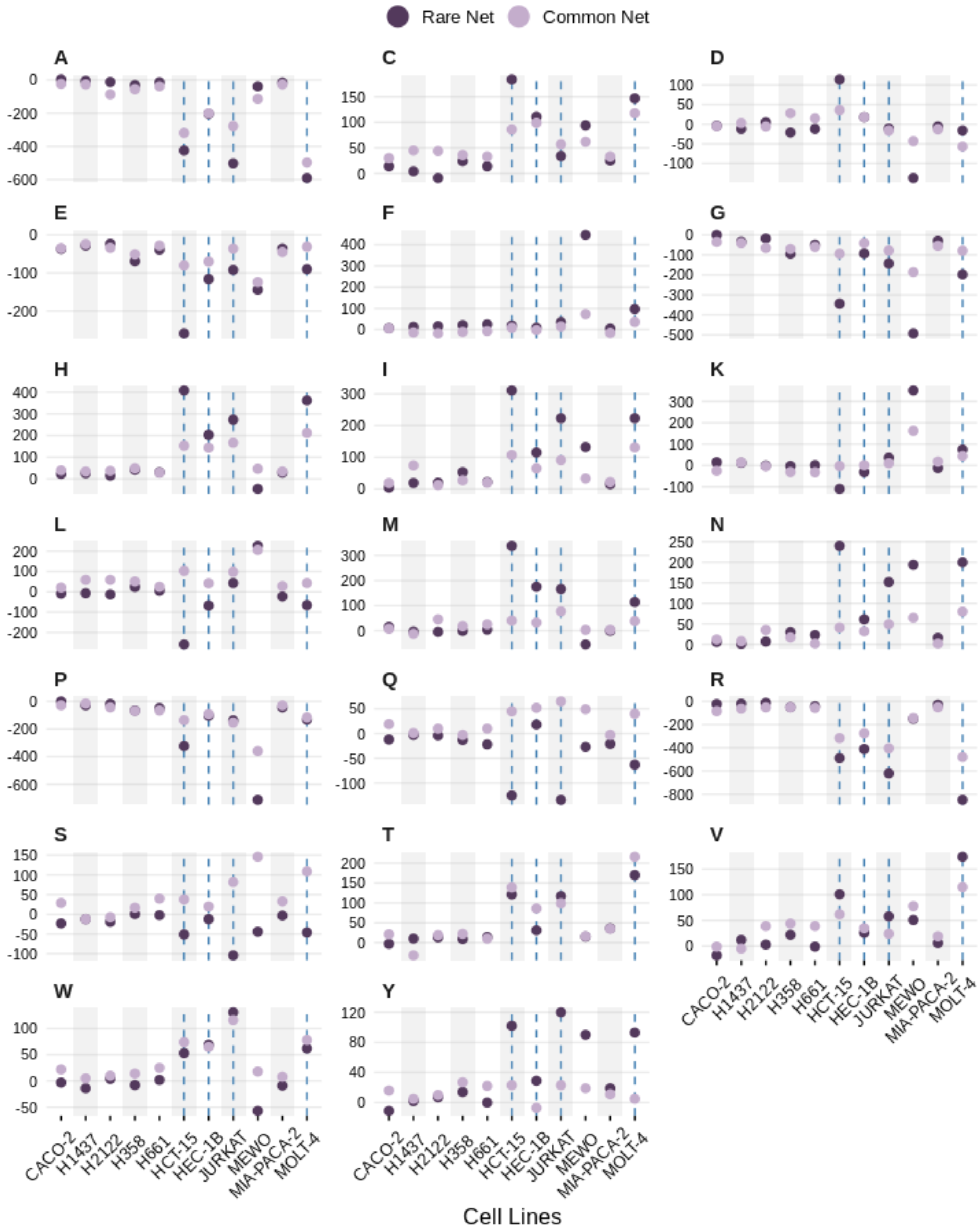

Supplementary Figure 12. Net amino acid counts in identified individual cell lines separated by common and rare. Dotted lines indicate dMMR cell lines. Source data are provided as a Source Data file. Letters correspond to the amino acid analyzed.

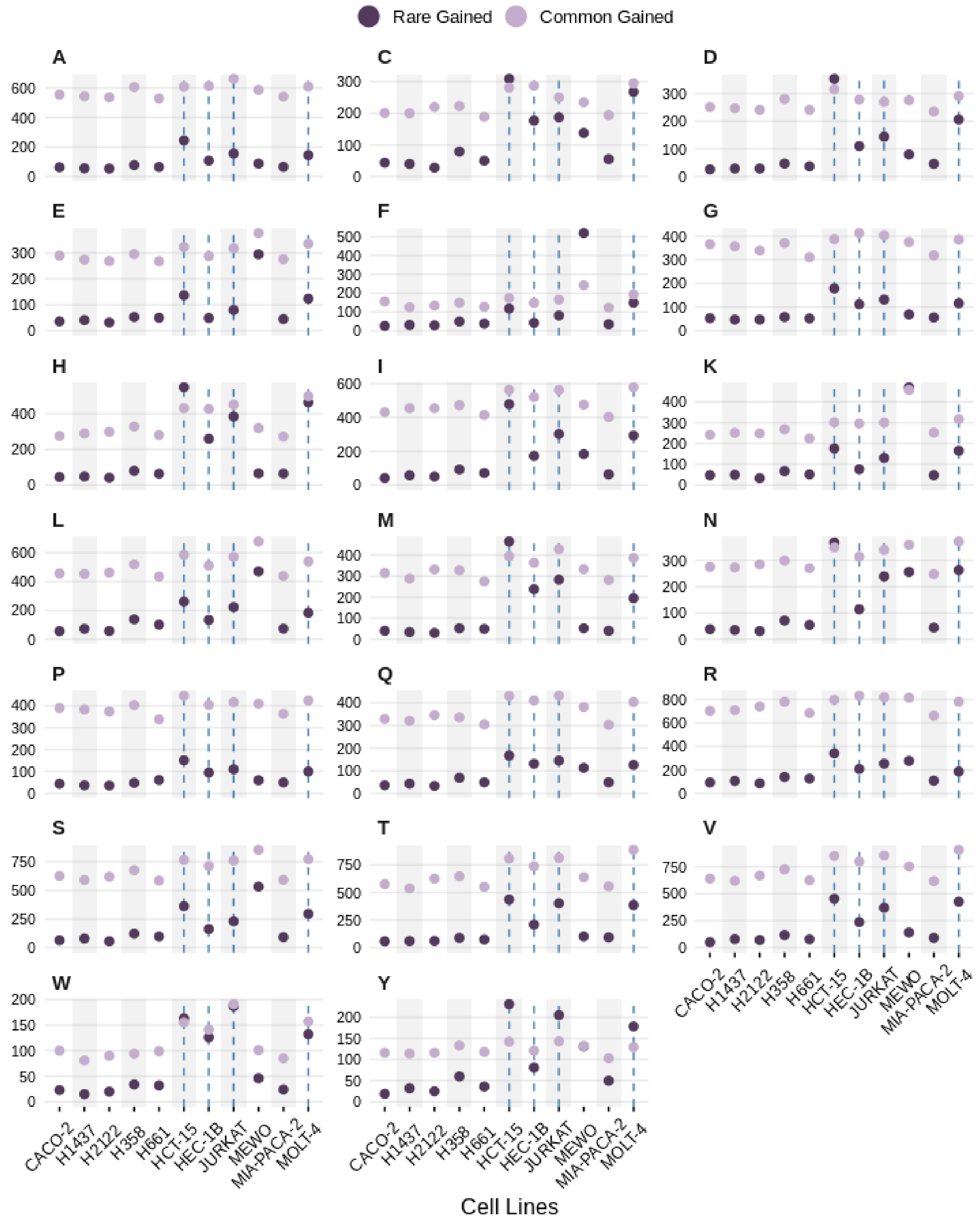

Supplementary Figure 13. Gained amino acid counts identified in individual cell lines separated by common and rare. Dotted lines indicate dMMR cell lines. Source data are provided as a Source Data file. Letters correspond to the amino acid analyzed.

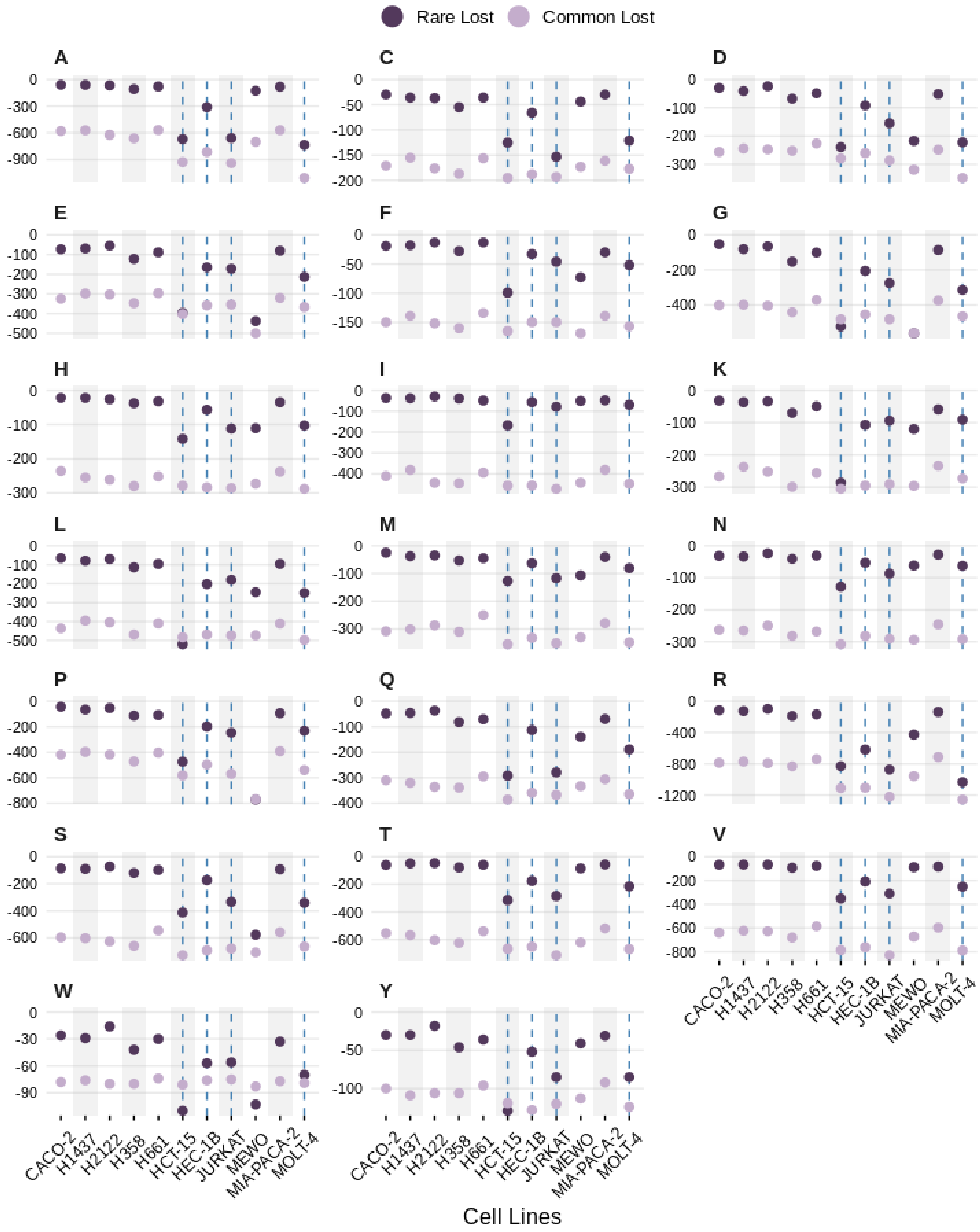

Supplementary Figure 14. Lost amino acid counts identified in individual cell lines separated by common and rare. Dotted lines indicate dMMR cell lines. Source data are provided as a Source Data file. Letters correspond to the amino acid analyzed.

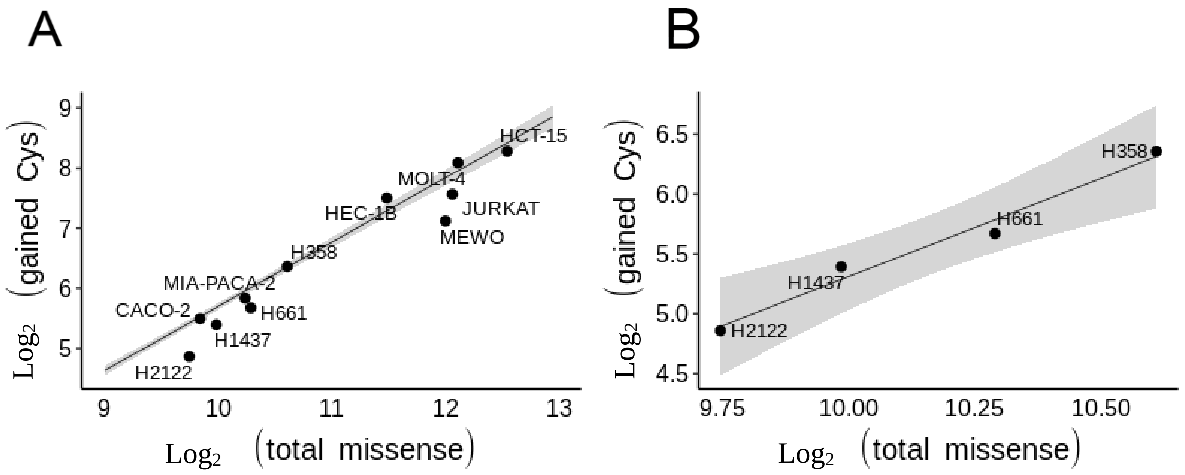

Supplementary Figure 15. A) Gained cysteine missense mutations relative to total missense mutations from sequencing data fit to all COSMIC-CLP linear model from Figure 2B and 2D. B) Lung cancer lines subset with linear regression and 95% confidence interval shaded in gray– suggests the comparatively modest impact of smoking on cysteine acquisition for the cell lines evaluated. Source data are provided as a Source Data file.

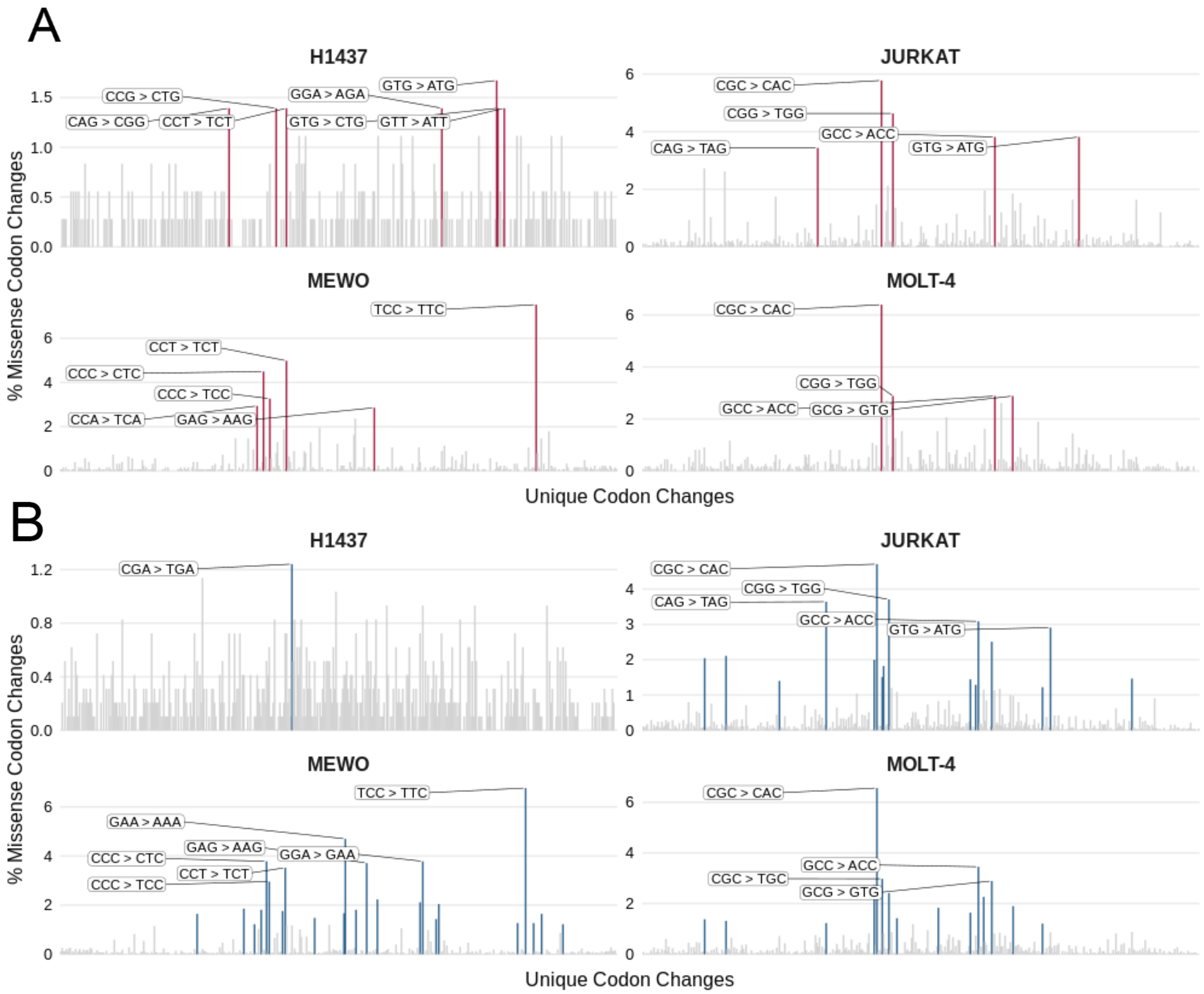

Supplementary Figure 16. Unique codon changes in Jurkat, NCI-H1437, MOLT-4 and MeWo cell lines; RNA changes in red and exome changes in blue; analysis of rare variants. Tobacco-smoke carcinogens, such as benzo[*a*]pyrene (B[*a*]P) diol epoxides, uniquely confer G→ T (C→ A) transversions that we see enriched only in our smoking LC line. dMMR and UV mutations are predominantly C→T (G→A) transitions, the flanking nucleotides differ as UV radiation induces pyrimidine dimers which largely result in CC→TT transitions (and counterpart GG→AA) which cause gain of F/K and loss of S/E. Source data are provided as a Source Data file.

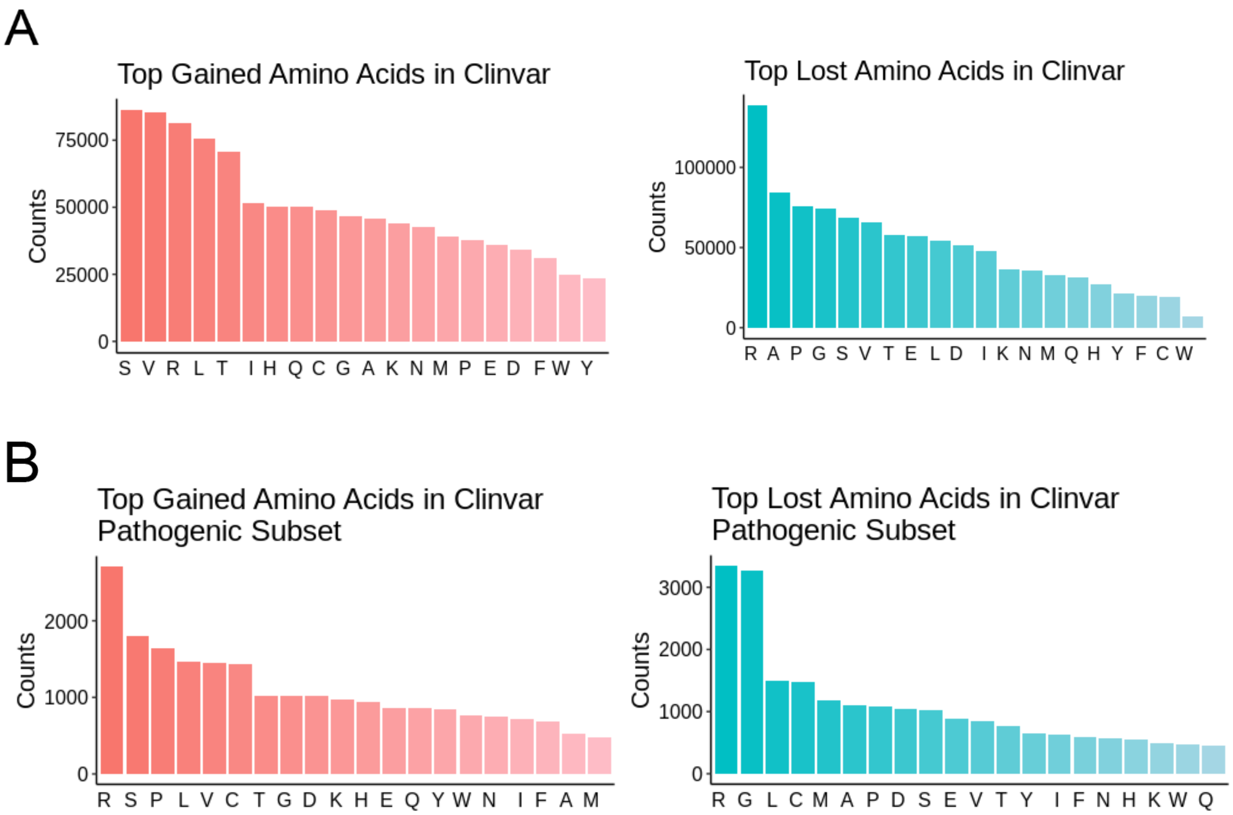

Supplementary Figure 17. ClinVar amino acid changes. A) Total gained and lost amino acid residues reported in all ClinVar data for unique gene name, protein position and amino acid change. B) Total gained and lost amino acids for pathogenic missense variants in ClinVar. Red indicates gained amino acid residues and blue indicates lost amino acid residues. Source data are provided as a Source Data file.

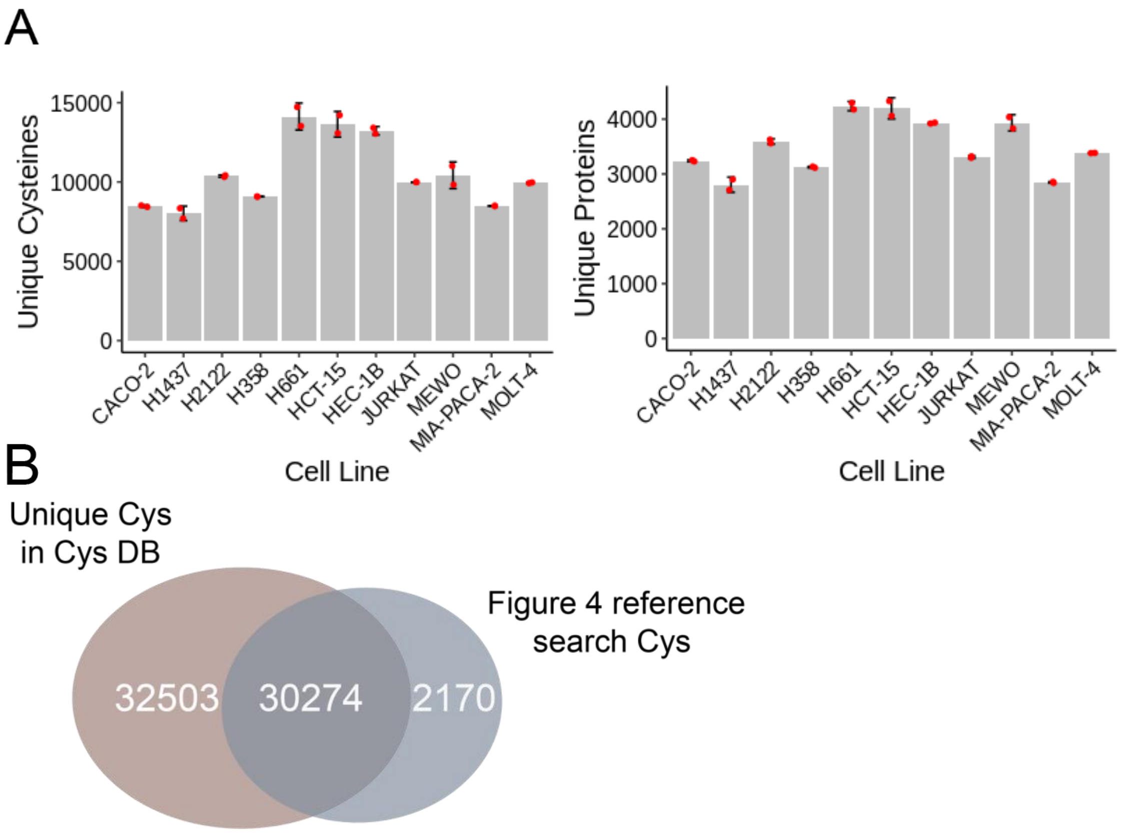

Supplementary Figure 18. A) Reference cysteines and proteins identified per cell line in Figure 4 and 6 proteomics for duplicate datasets (n=2). B) Figure 4 and 6 proteomics reference cysteines overlap with CysDB cysteines. Red indicates Cys DB dataset, blue indicates Figure 4 and 6 proteomics dataset. Showing mean values and error bars = SD. Source data are provided as a Source Data file.

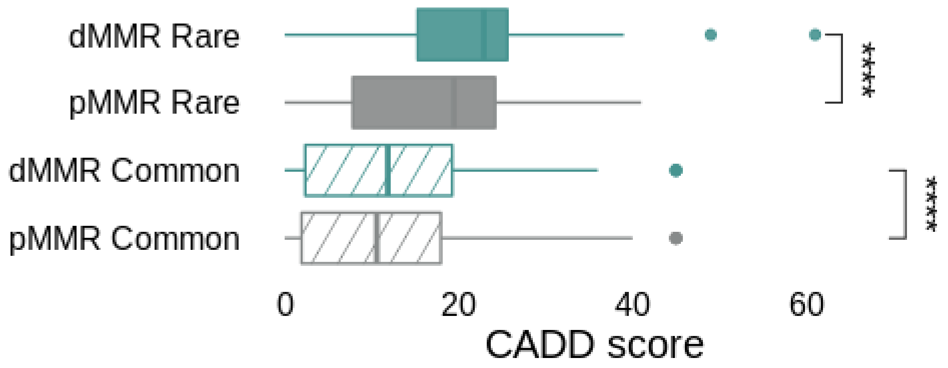

Supplementary Figure 19. Distribution of CADD (Combined Annotation Dependent Depletion) scores for indicated variant grouping from Figure 3 and 5 genomic data, Statistical significance was calculated using two-sided Mann-Whitney U test, **** p < 0.0001. Box plot center line, median; limits are upper and lower quartiles; 1.5x interquartile range. Source data are provided as a Source Data file.

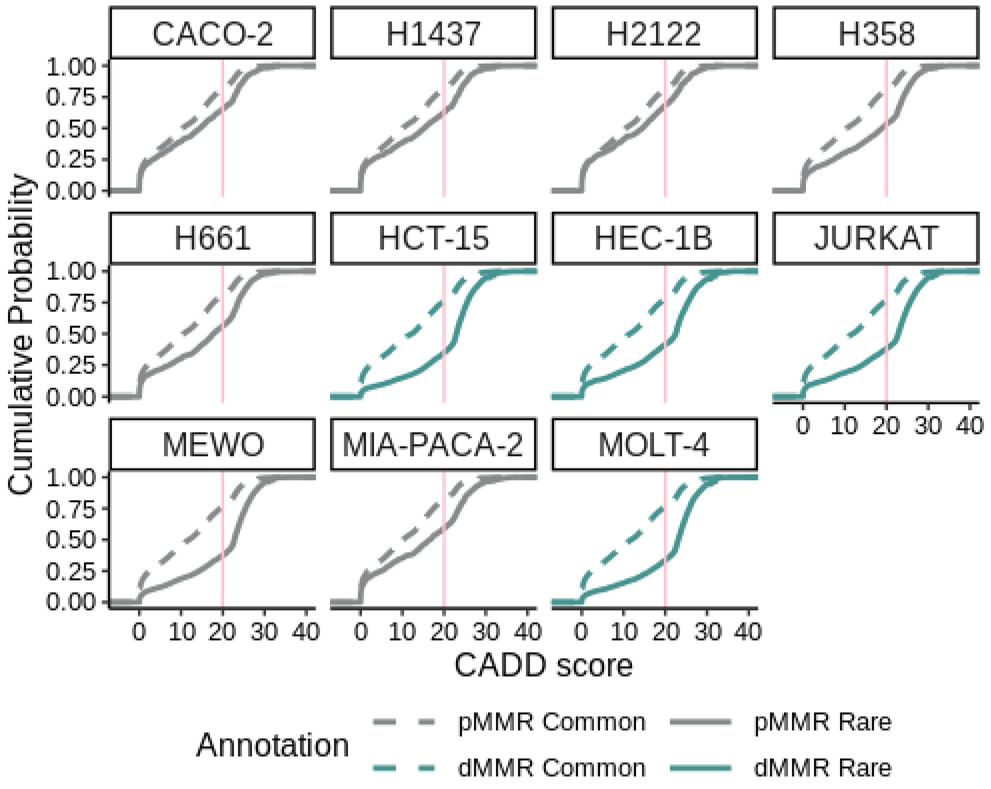

Supplementary Figure 20. CADD-phred cumulative probabilities stratified by cell line from Figure 3 and 5 genomic data. Pink lines indicate CADD-phred = 20. Source data are provided as a Source Data file.

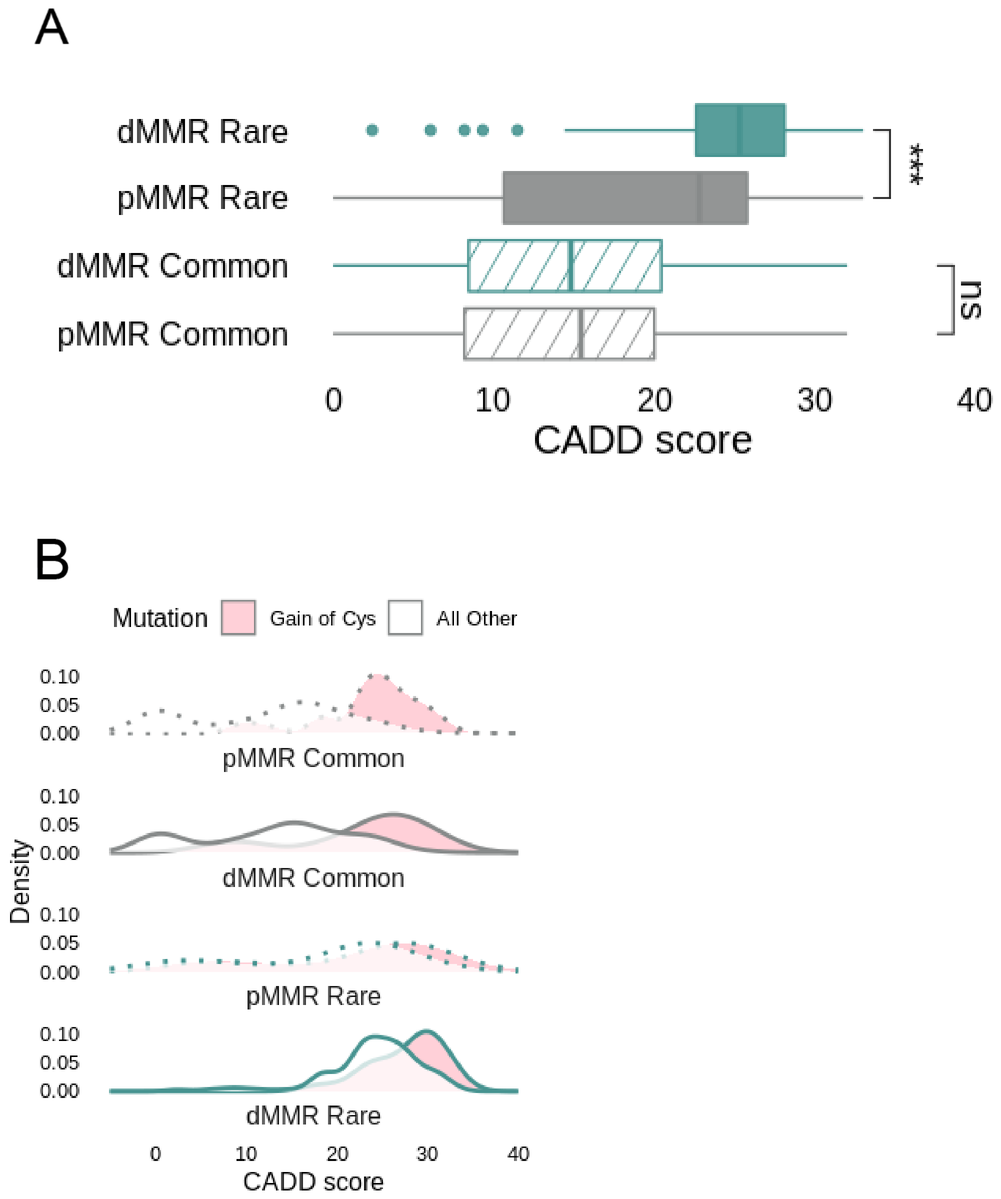

Supplementary Figure 21. A) Distribution of CADD-phred scores for indicated variant grouping from Figure 3 and 5 proteomic data. Statistical significance was calculated using two-sided Mann-Whitney U test, *** $p < 0.001$. Box plot center line, median; limits are upper and lower quartiles; 1.5x interquartile range. B) Distribution of CADD-phred scores for indicated gaind cysteines vs all other from Figure 3 and 5 proteomic data. Source data are provided as a Source Data file.

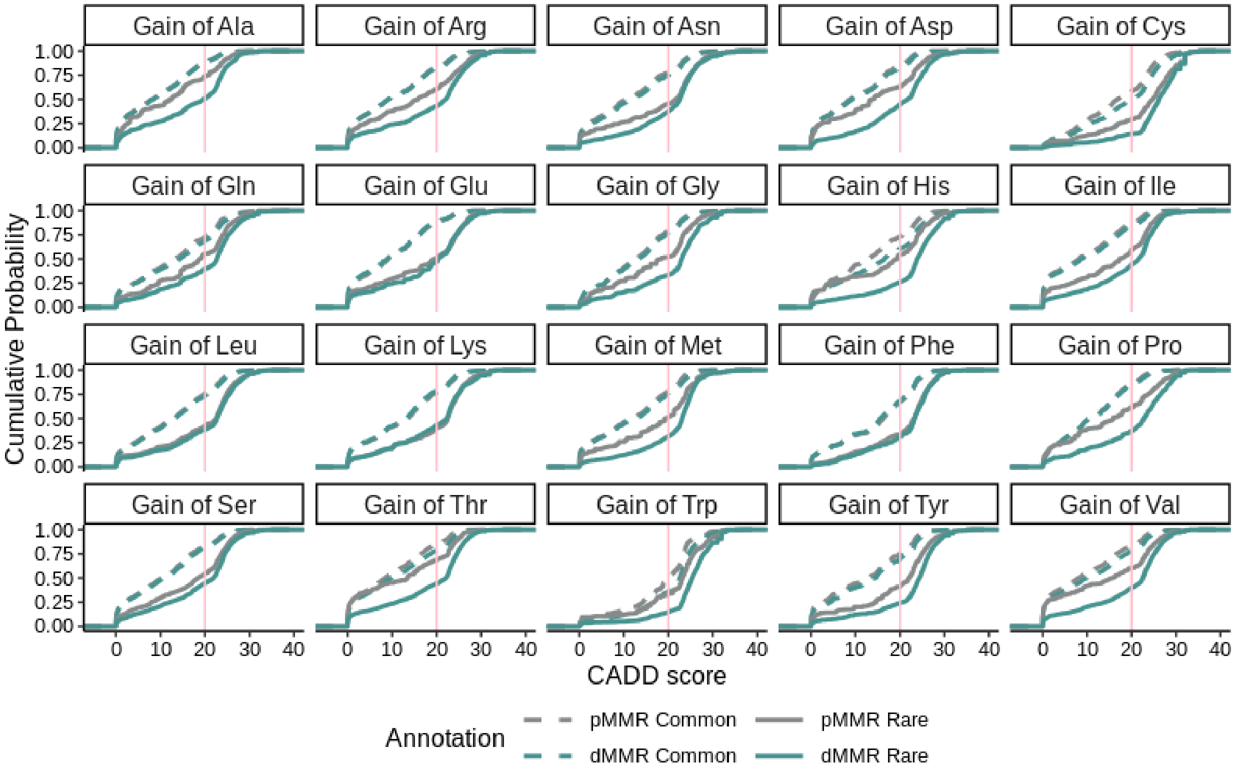

Supplementary Figure 22. CADD-phred cumulative probabilities stratified by gained amino acid from genomics data. Pink lines indicate CADD-phred = 20. One sided Mann Whitney U test p-values are in Supplementary Data S3 (tab 28). Source data are provided as a Source Data file.

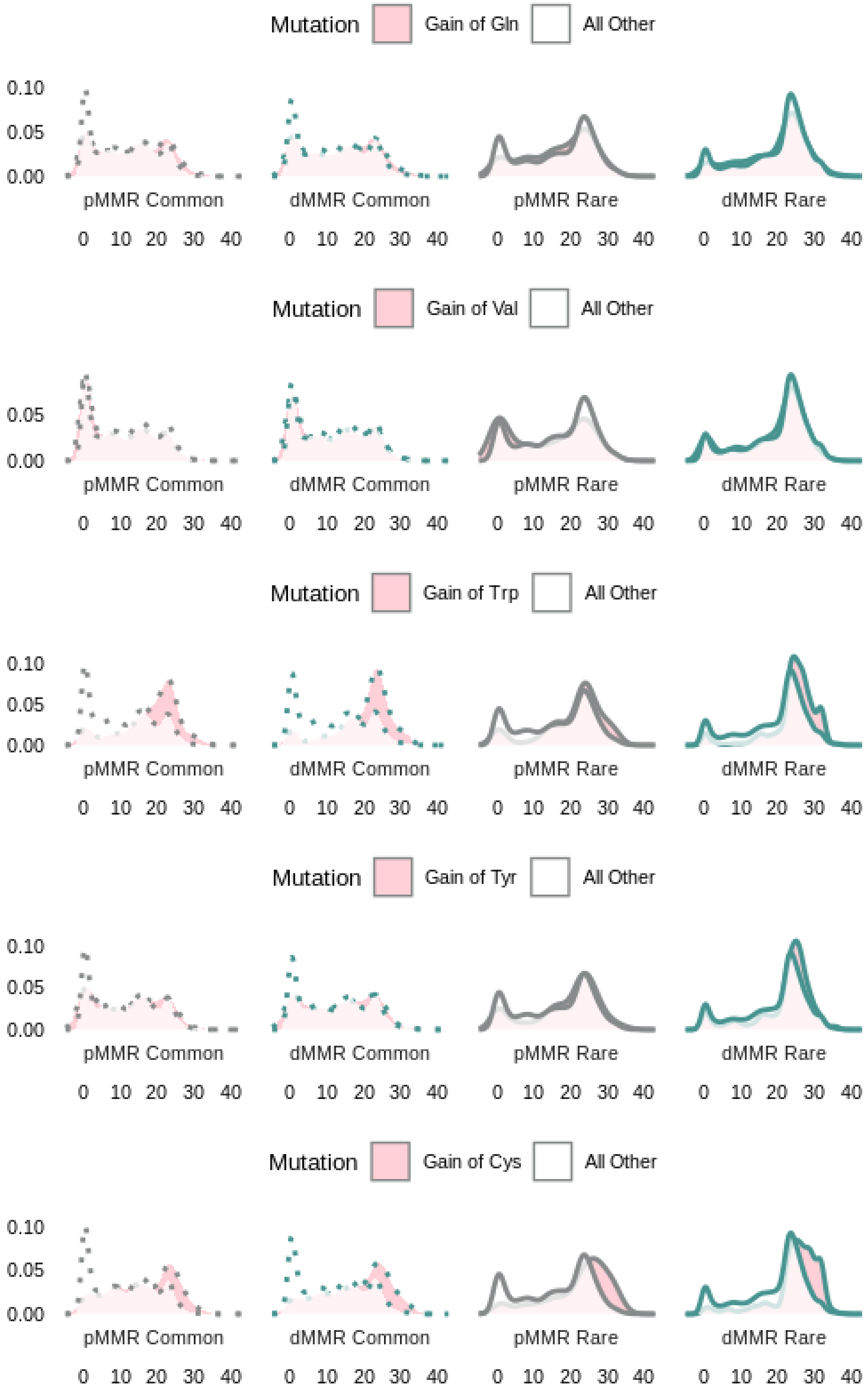
Mutation
Gain of Gln
All Other
0.10
0.05
0.00
pMMR Common
dMMR Common
pMMR Rare
dMMR Rare
0 10 20 30 40
Mutation
Gain of Val
All Other
0.05
0.00
pMMR Common
dMMR Common
pMMR Rare
dMMR Rare
0 10 20 30 40
Mutation
Gain of Trp
All Other
0.10
0.05
0.00
pMMR Common
dMMR Common
pMMR Rare
dMMR Rare
0 10 20 30 40
Mutation
Gain of Tyr
All Other
0.10
0.05
0.00
pMMR Common
dMMR Common
pMMR Rare
dMMR Rare
0 10 20 30 40
Mutation
Gain of Cys
All Other
0.10
0.05
0.00
pMMR Common
dMMR Common
pMMR Rare
dMMR Rare
0 10 20 30 40

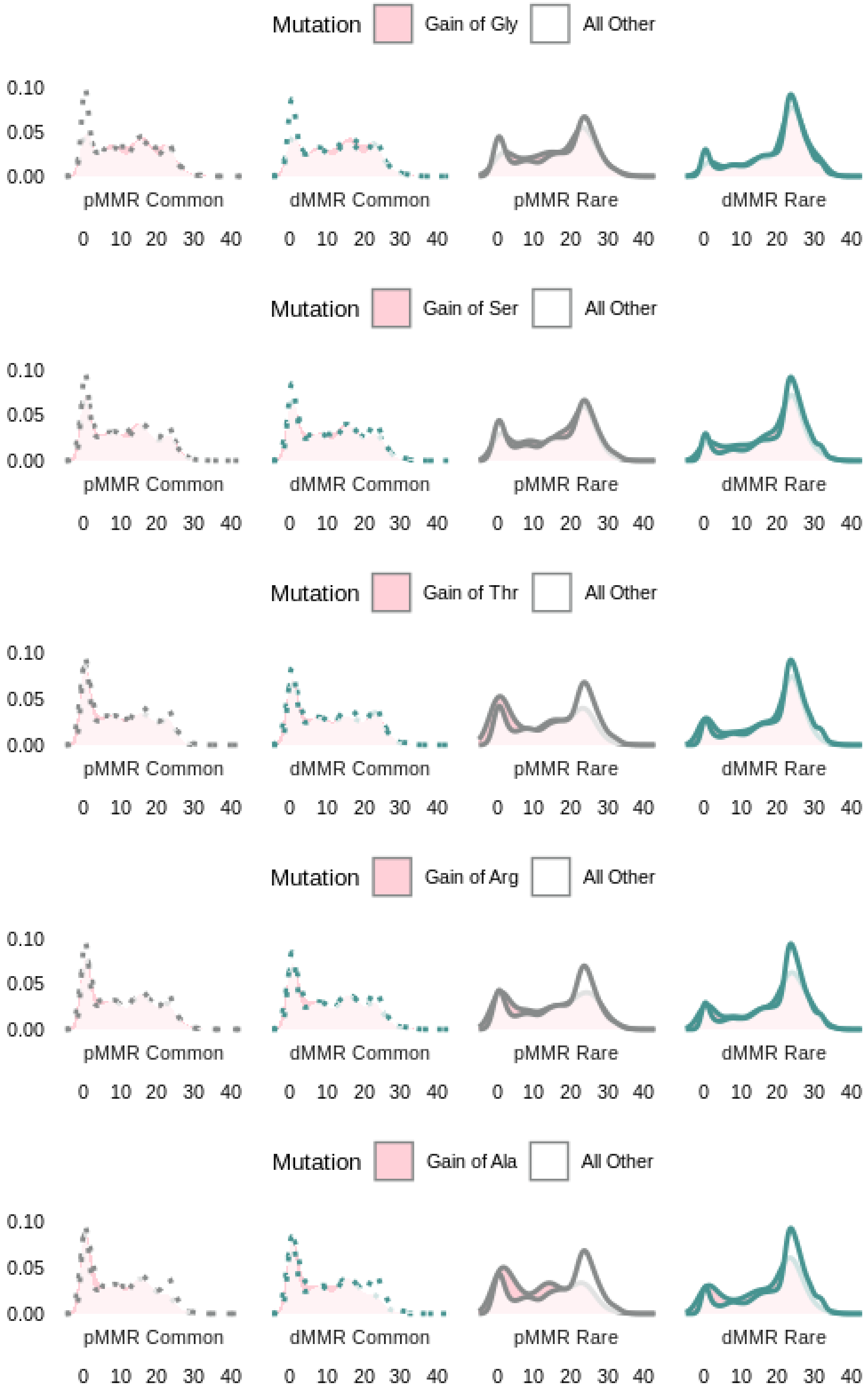
Mutation
Gain of Gly
All Other
0.10
0.05
0.00
pMMR Common
dMMR Common
pMMR Rare
dMMR Rare
0 10 20 30 40
Mutation
Gain of Ser
All Other
Mutation
Gain of Thr
All Other
Mutation
Gain of Arg
All Other
Mutation
Gain of Ala
All Other

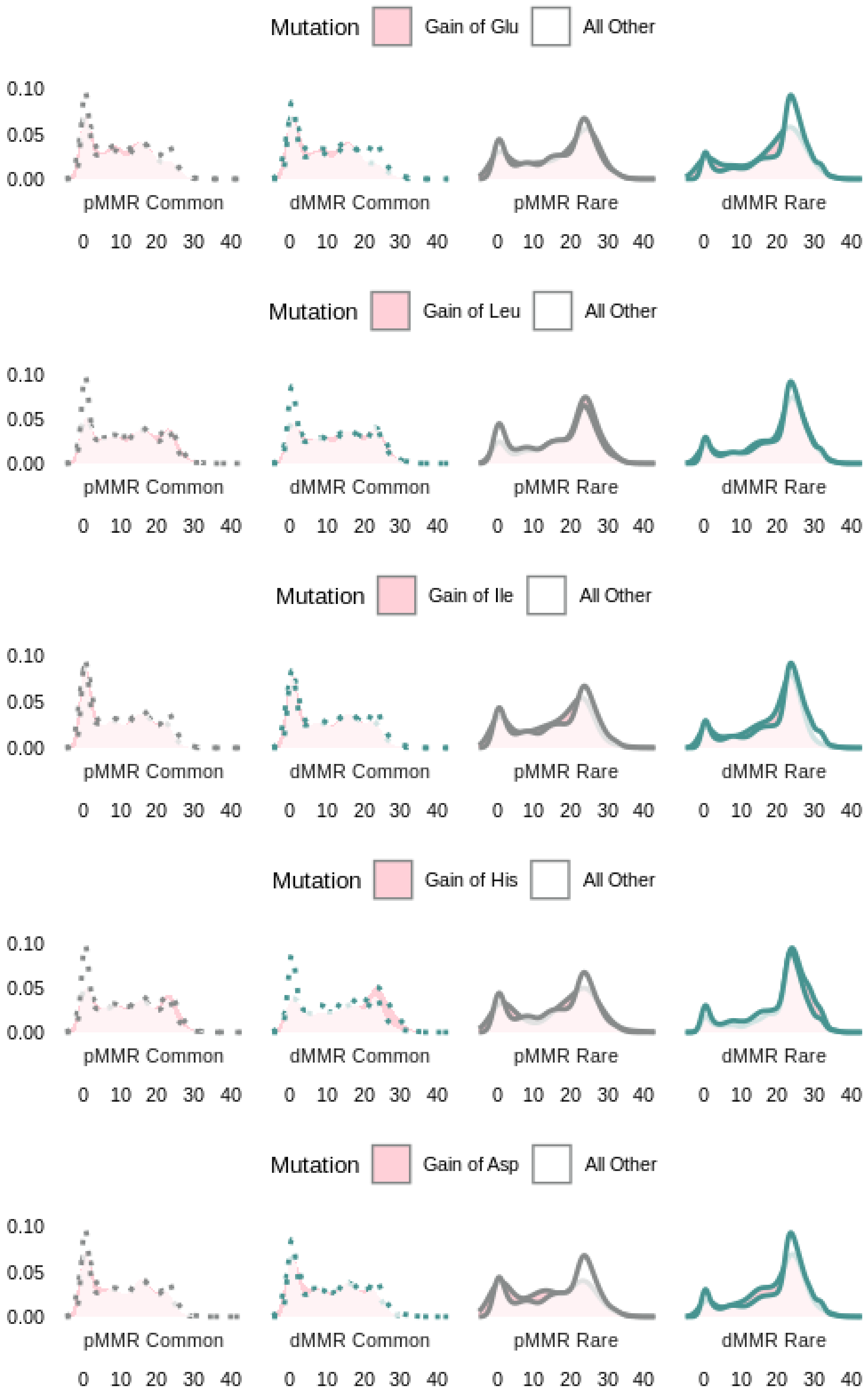
Mutation
Gain of Glu
All Other
Mutation
Gain of Leu
All Other
Mutation
Gain of Ile
All Other
Mutation
Gain of His
All Other
Mutation
Gain of Asp
All Other
0.10
0.05
0.00
pMMR Common
dMMR Common
pMMR Rare
dMMR Rare
0 10 20 30 40

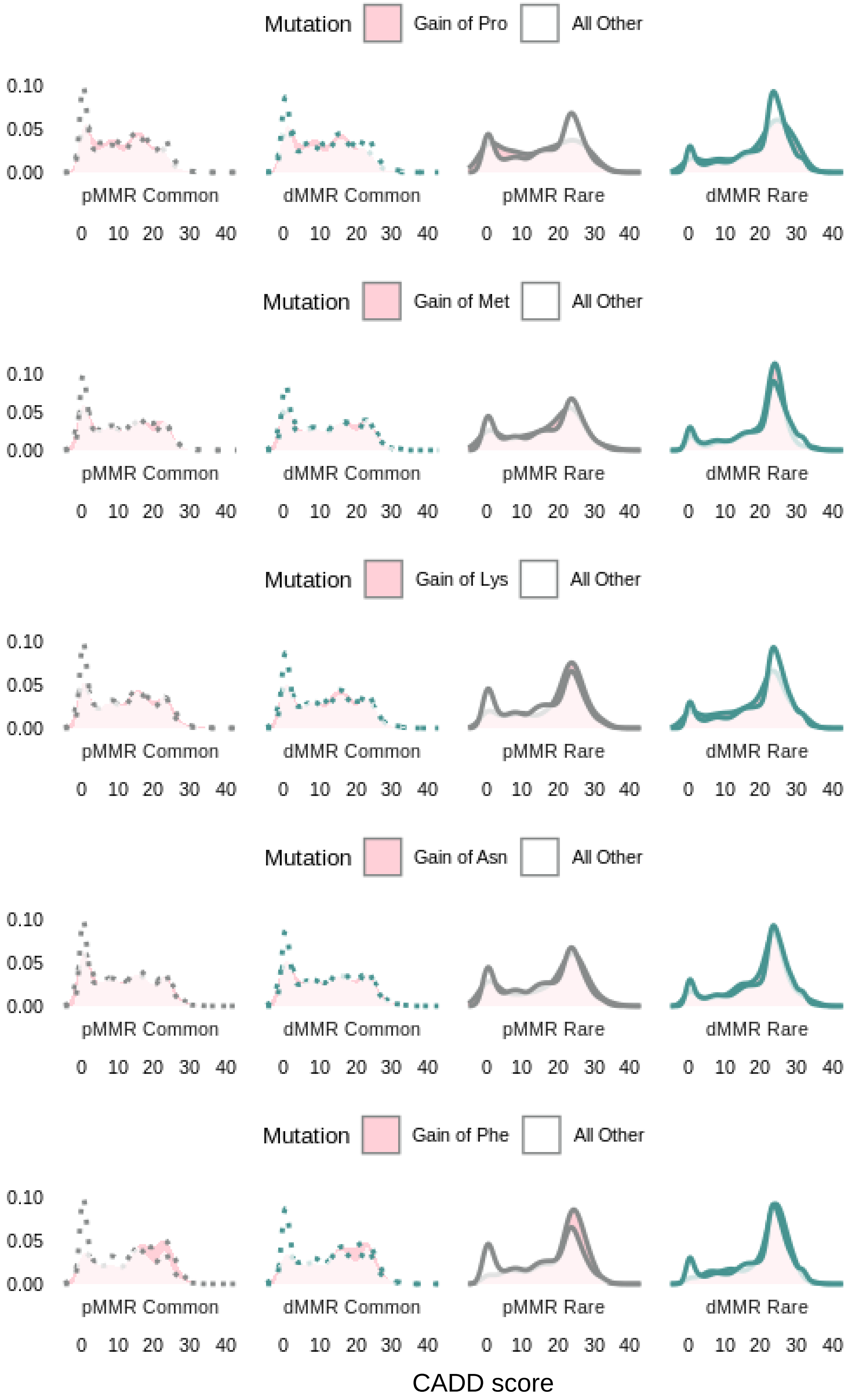

Supplementary Figure 23. Distribution of CADD-phred scores for indicated amino-acid gained

grouping. One sided Mann Whitney U test p-values are in Supplementary Data S3 (tab 28). Source data are provided as a Source Data file.

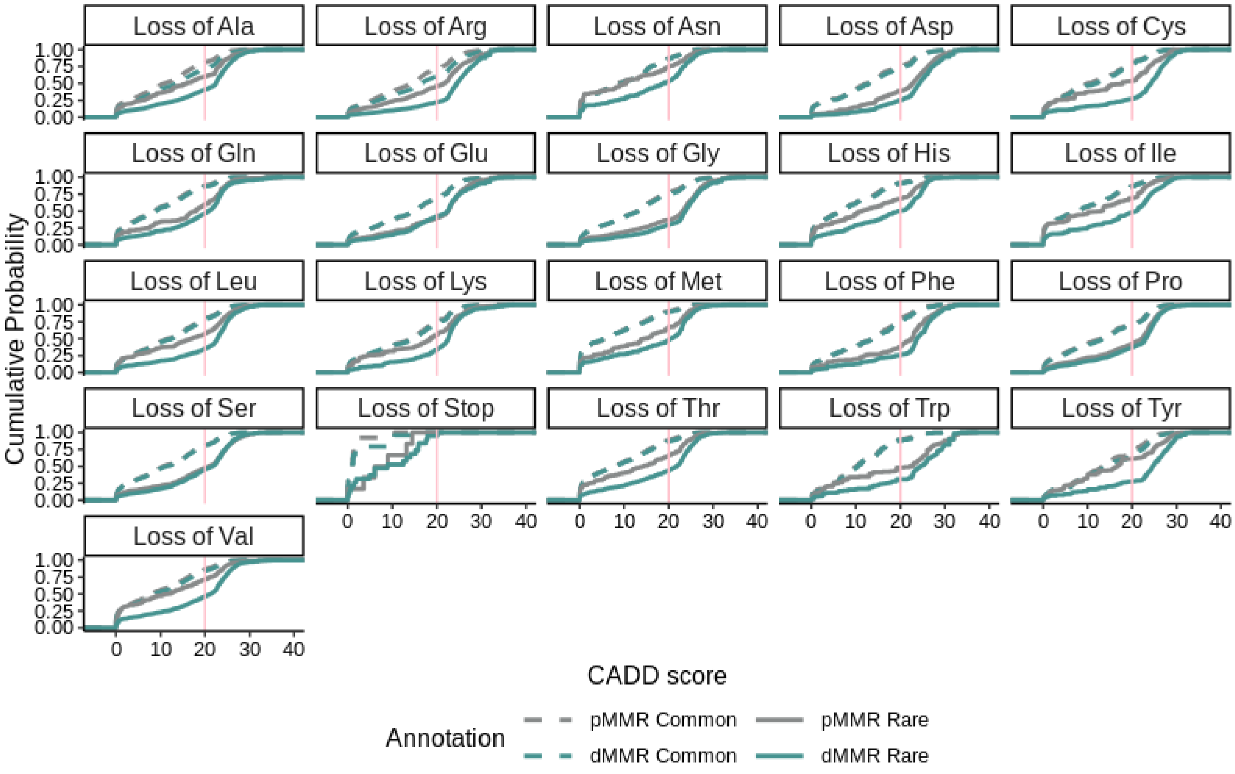

Supplementary Figure 24. CADD-phred cumulative probabilities stratified by lost amino acid residues. Pink lines indicate CADD-phred = 20. One sided Mann Whitney U test p-values are in Supplementary Data S3 (tab 28). Source data are provided as a Source Data file.

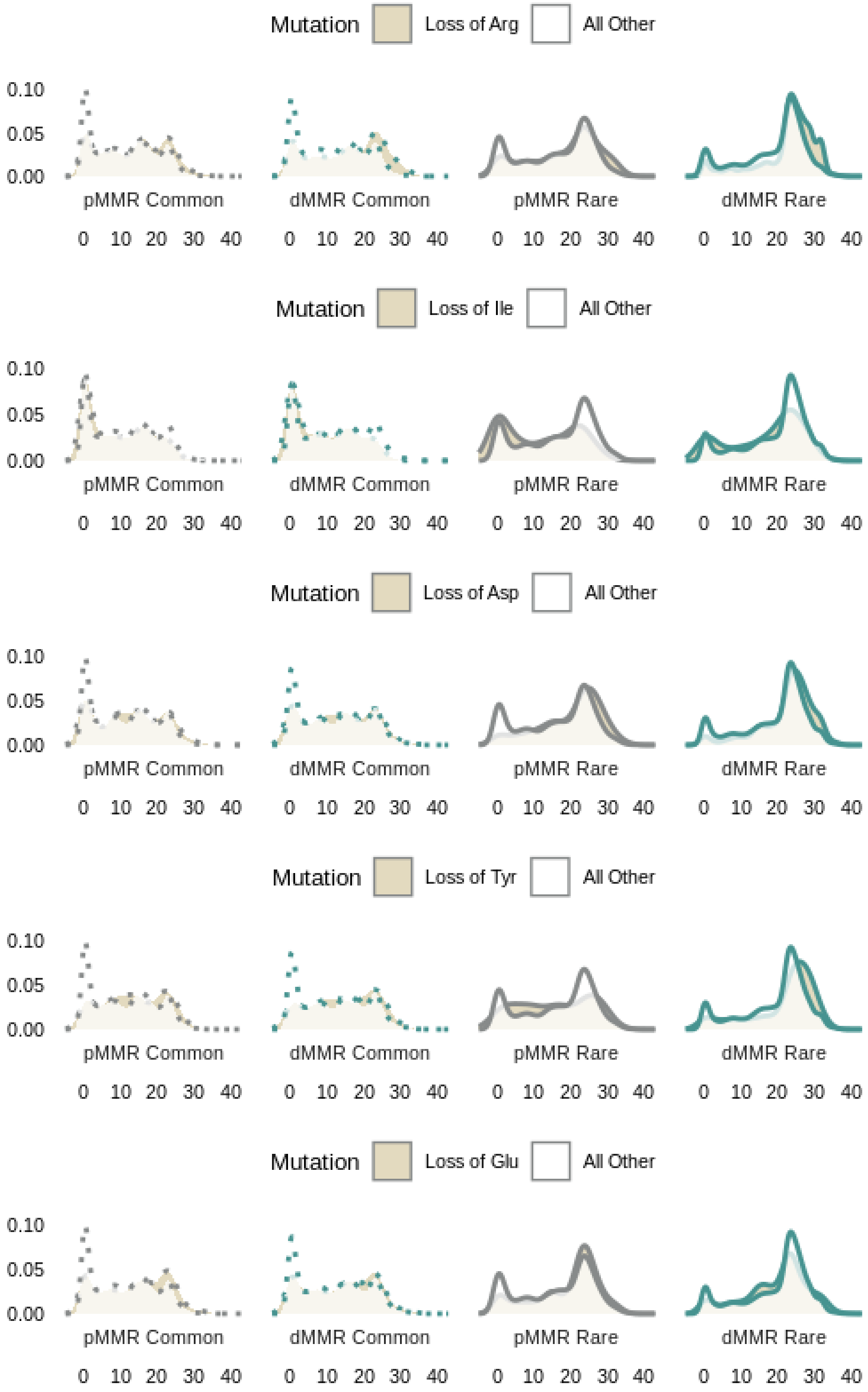
Mutation
Loss of Arg
All Other
0.10
0.05
0.00
pMMR Common
dMMR Common
pMMR Rare
dMMR Rare
0 10 20 30 40
Mutation
Loss of Ile
All Other
0.10
0.05
0.00
pMMR Common
dMMR Common
pMMR Rare
dMMR Rare
0 10 20 30 40
Mutation
Loss of Asp
All Other
0.10
0.05
0.00
pMMR Common
dMMR Common
pMMR Rare
dMMR Rare
0 10 20 30 40
Mutation
Loss of Tyr
All Other
0.10
0.05
0.00
pMMR Common
dMMR Common
pMMR Rare
dMMR Rare
0 10 20 30 40
Mutation
Loss of Glu
All Other
0.10
0.05
0.00
pMMR Common
dMMR Common
pMMR Rare
dMMR Rare
0 10 20 30 40

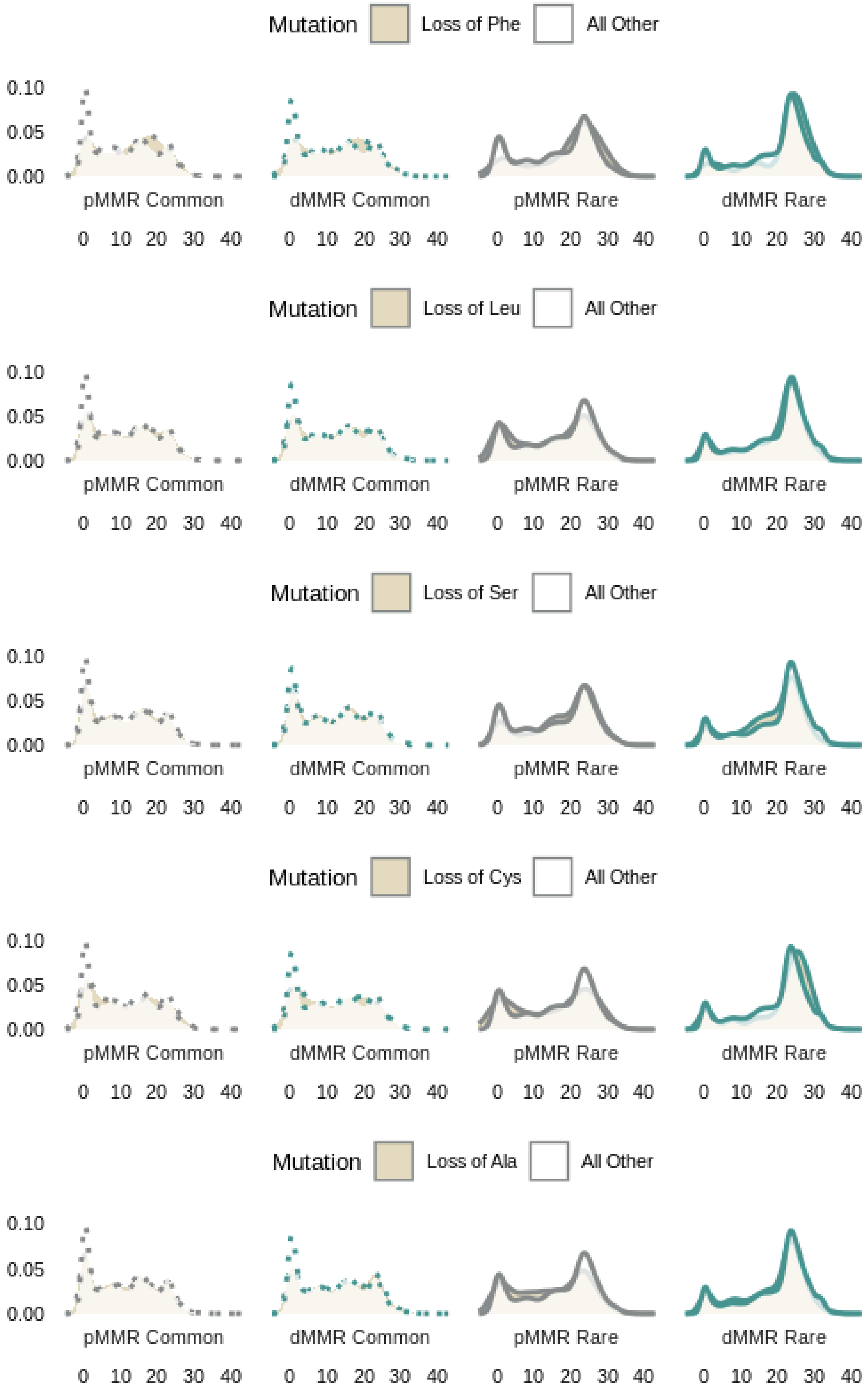
Mutation
Loss of Phe
All Other
Loss of Leu
Loss of Ser
Loss of Cys
Loss of Ala
0.10
0.05
0.00
pMMR Common
dMMR Common
pMMR Rare
dMMR Rare
0 10 20 30 40

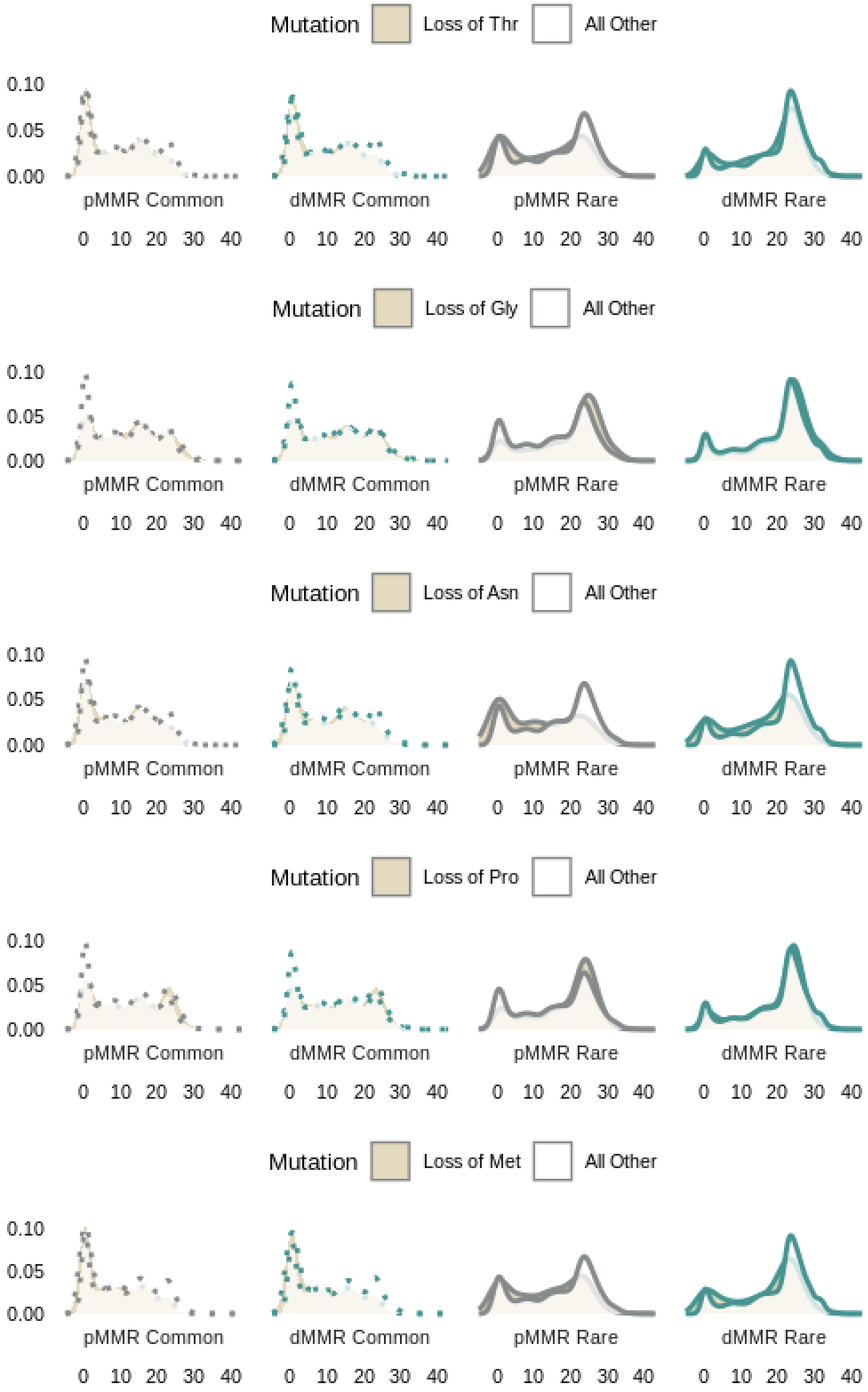
Mutation
Loss of Thr
All Other
0.10
0.05
0.00
pMMR Common
dMMR Common
pMMR Rare
dMMR Rare
0 10 20 30 40
Mutation
Loss of Gly
All Other
Mutation
Loss of Asn
All Other
Mutation
Loss of Pro
All Other
Mutation
Loss of Met
All Other

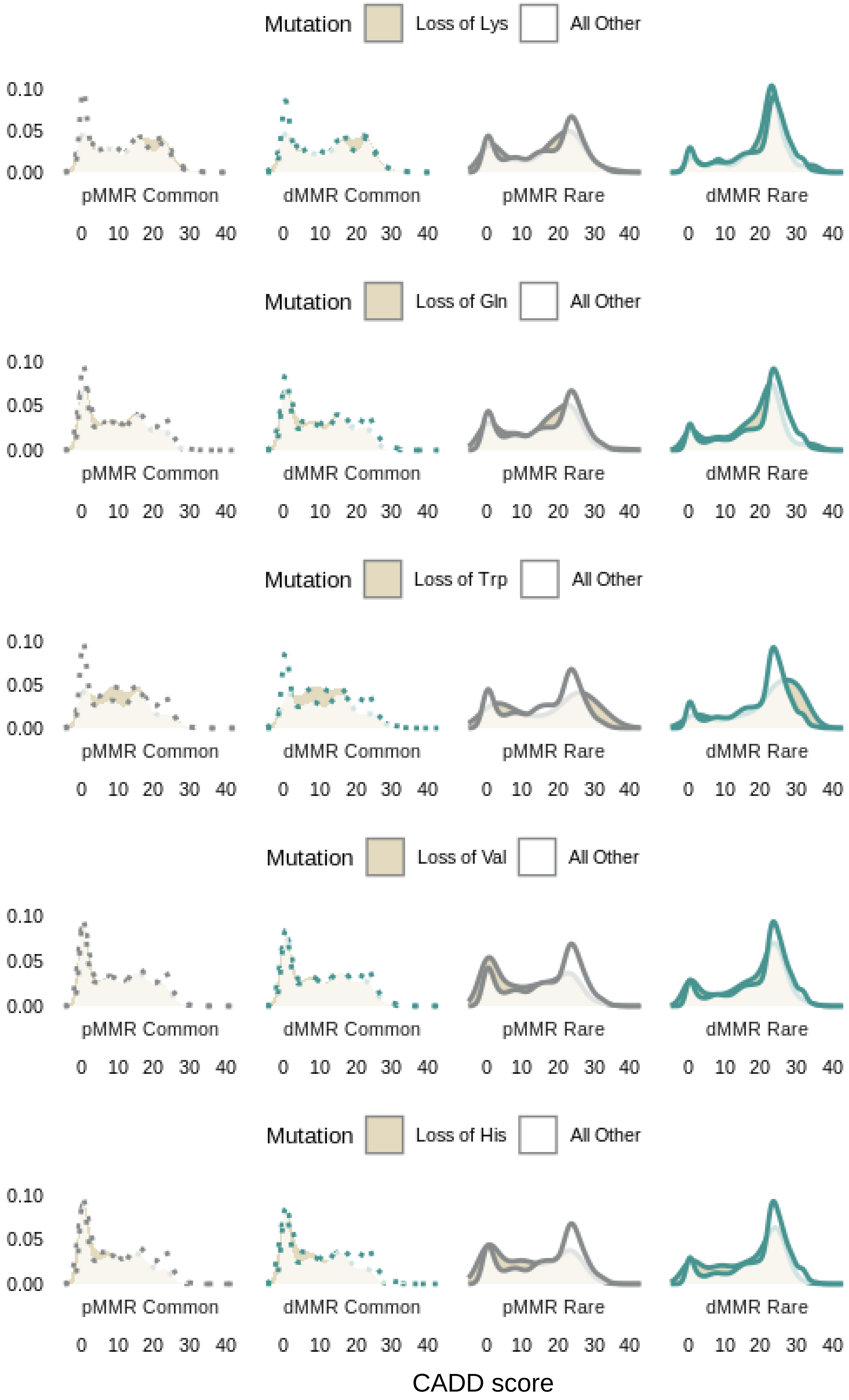

Supplementary Figure 25. Distribution of CADD-phred scores for indicated amino-acid loss grouping. One sided Mann Whitney U test p-values are in Supplementary Data S3 (tab 28).

Source data are provided as a Source Data file.

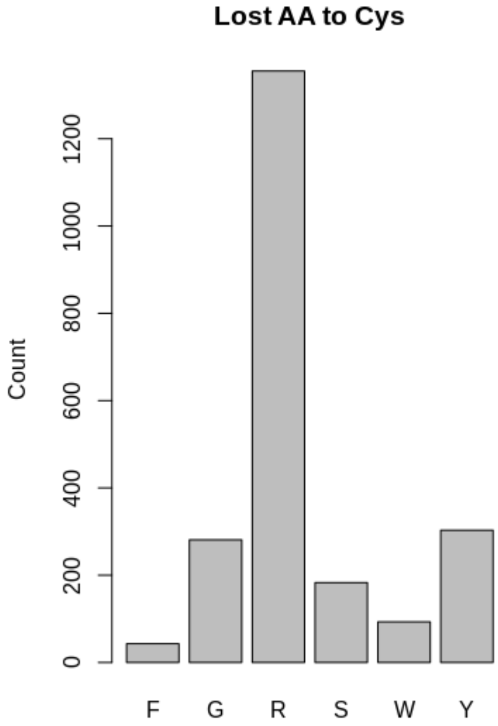

Supplementary Figure 26. Distribution of reference/lost amino acids of gained cysteines in Figure 3 and 5 genomic data (60% arginine). Source data are provided as a Source Data file.

A

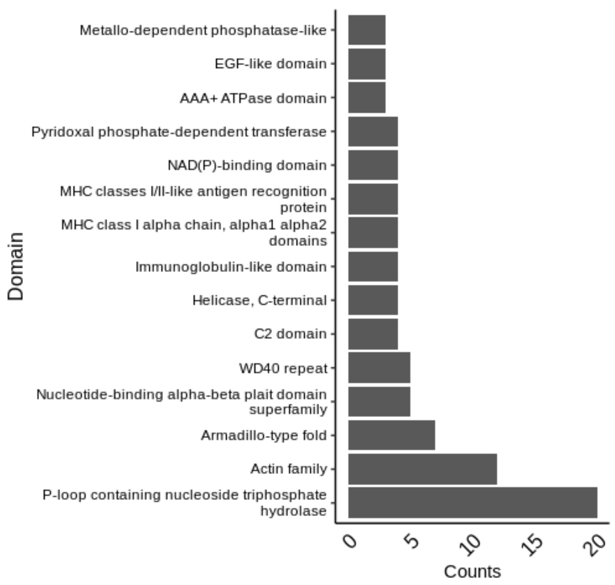

Chemoproteomics detected variant domains

B

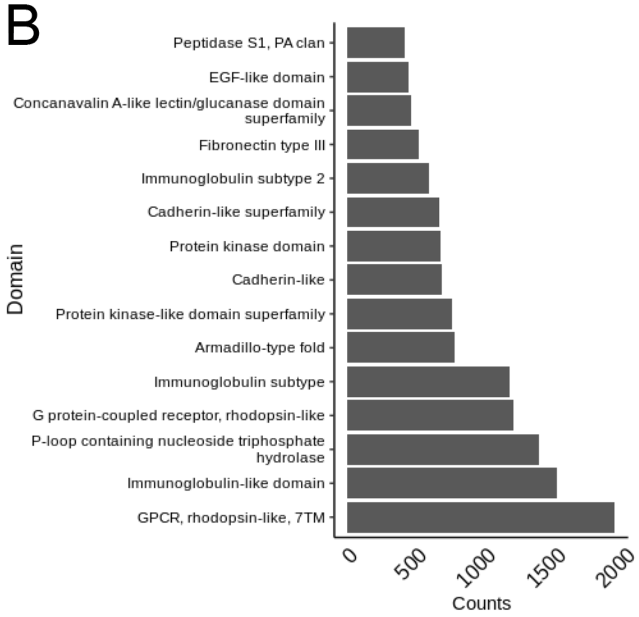

Genomics detected variant domains

Supplementary Figure 27. A) Identified variants' domain residence counts from Figure 3 and 5 proteomics data B) Identified variants' domain residence counts from Figure 3 and 5 genomics data. Source data are provided as a Source Data file.

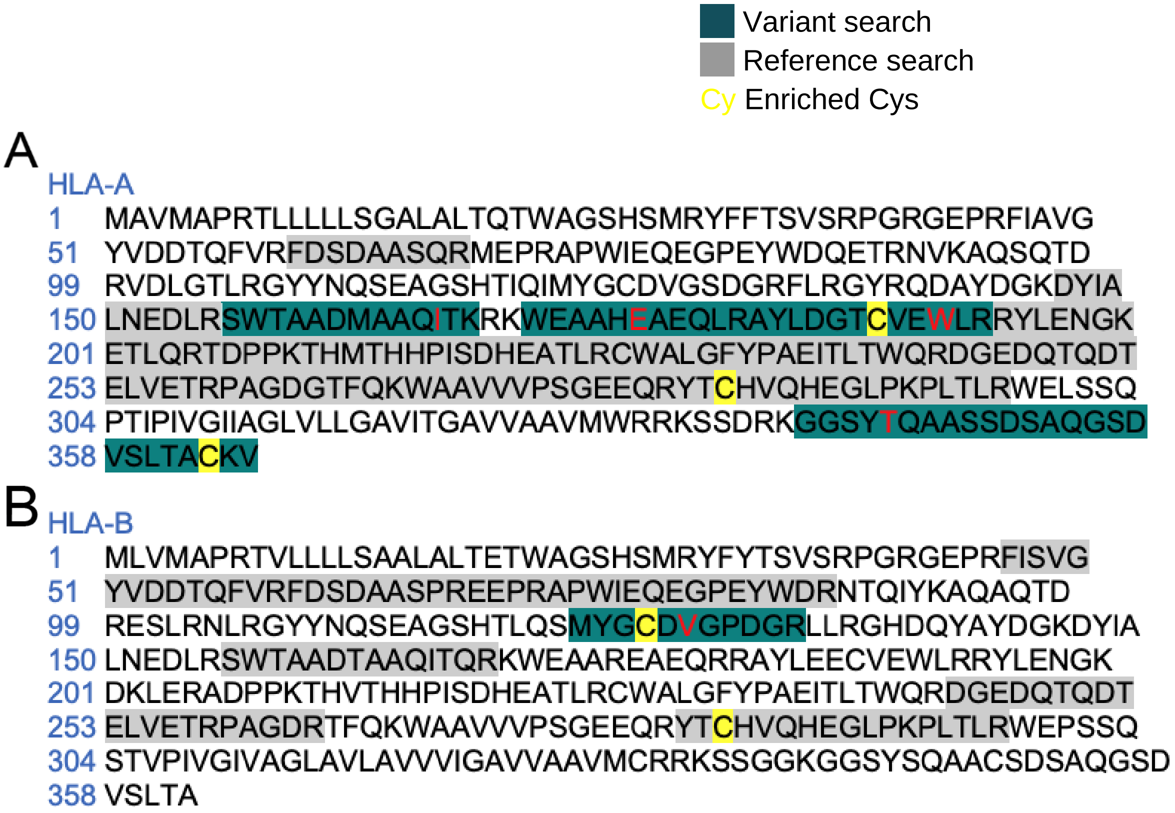

Supplementary Figure 28. Sequence coverage of Uniprot references A) HLA-A*3:01 and B) HLA-B*7:02 peptides from HCT-15 reference and variant searches in Figure 3-8 proteomics datasets; yellow indicates enriched cysteines and red are variant sites.

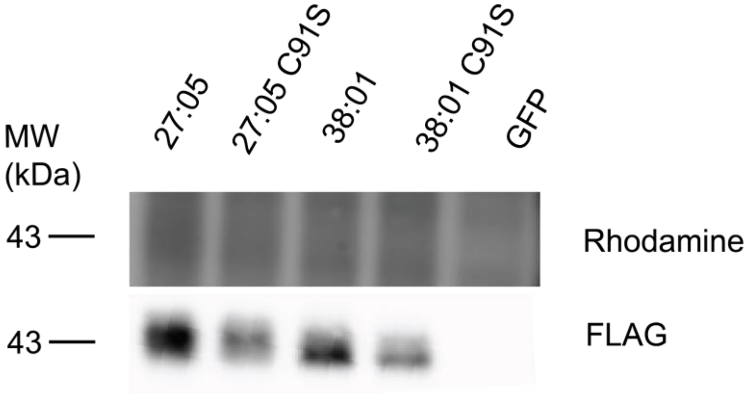

Supplementary Figure 29. Cysteine lysate labeling of overexpressed HLA-B alleles with iodoacetamide alkyne (IAA) (representative of 2 two biological replicates) conjugation by click chemistry to a rhodamine-azide tag described in methods. Source data are provided as a Source Data file.

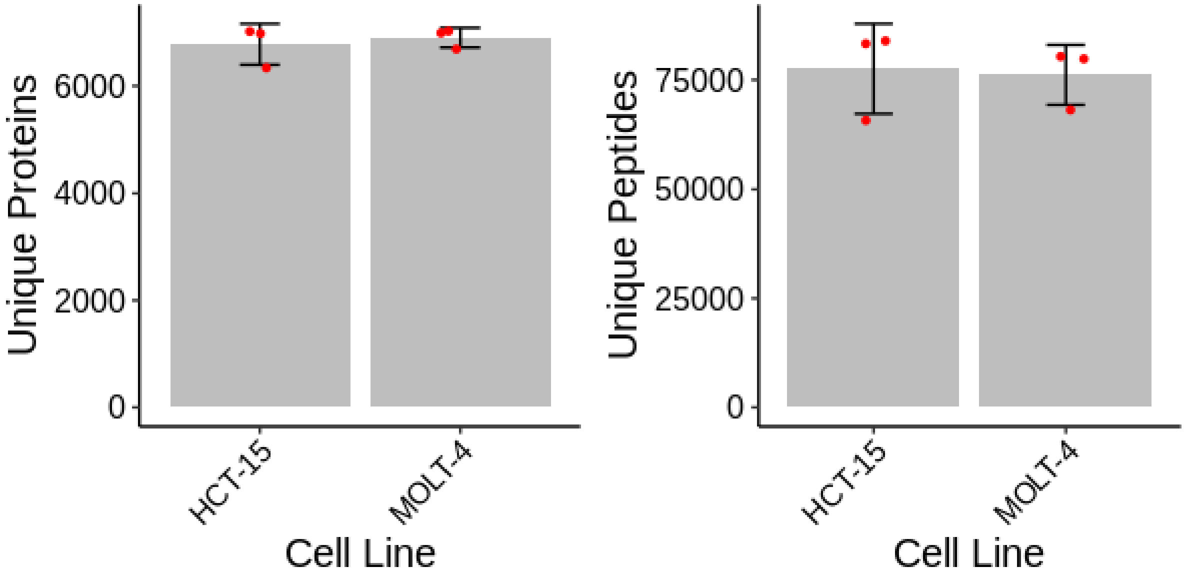

Supplementary Figure 30. Reference peptides and proteins identified per cell line in Figure 7 for triplicate sets of high pH fractionated samples per cell line. Showing mean values and error bars = SD. Source data are provided as a Source Data file.

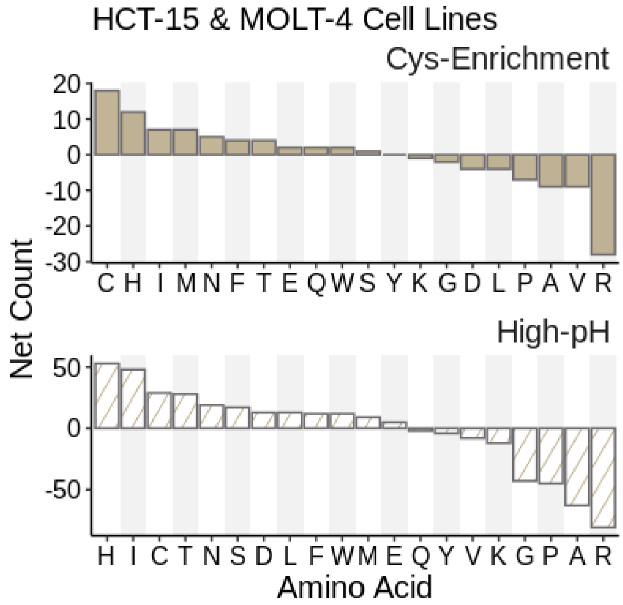

Supplementary Figure 31. Net counts of SAAVs identified by proteomics in Figure 7. Source data are provided as a Source Data file.

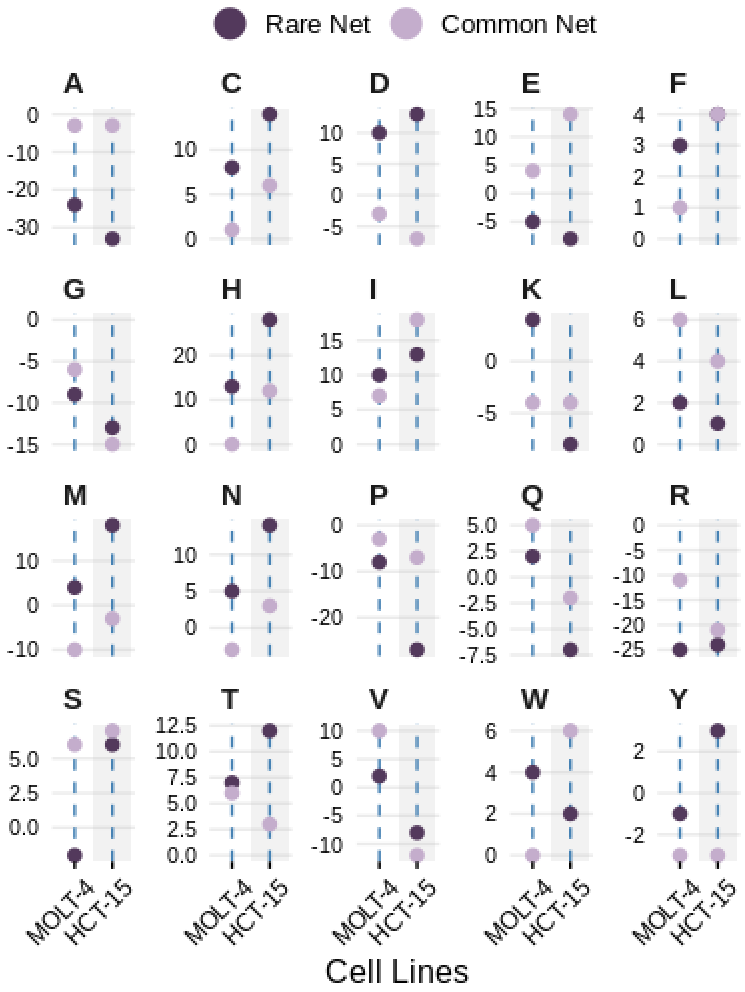

Supplementary Figure 32. Net counts of SAAVs identified by chemoproteomics stratified by amino acid and common vs rare identified in Figure 7. Source data are provided as a Source Data file. Letters correspond to the amino acid analyzed.

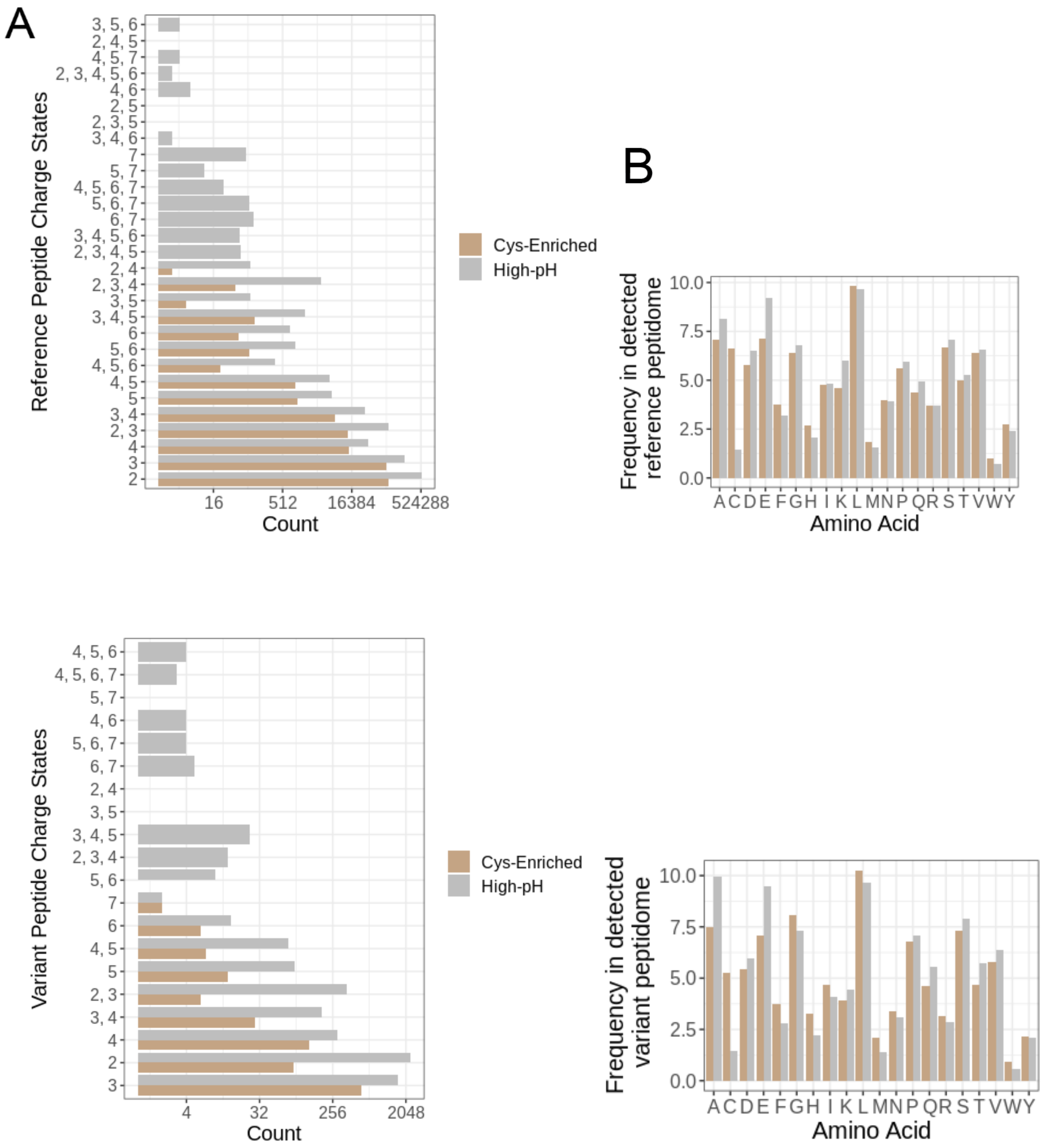

Supplementary Figure 33: Peptide properties of detected reference peptides from cys-enrichment and high-pH fractionation. A) Charge states of detected reference peptides and variant peptides. B) Abundance of amino acids in detected reference and variant peptides. Source data are provided as a Source Data file.

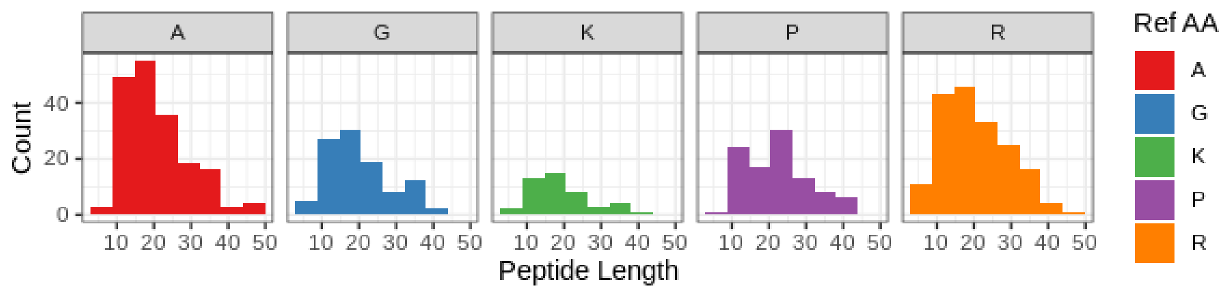

Supplementary Figure 34: High-pH identified variant peptide length and SAAV reference amino acid for top lost amino acids. Source data are provided as a Source Data file.

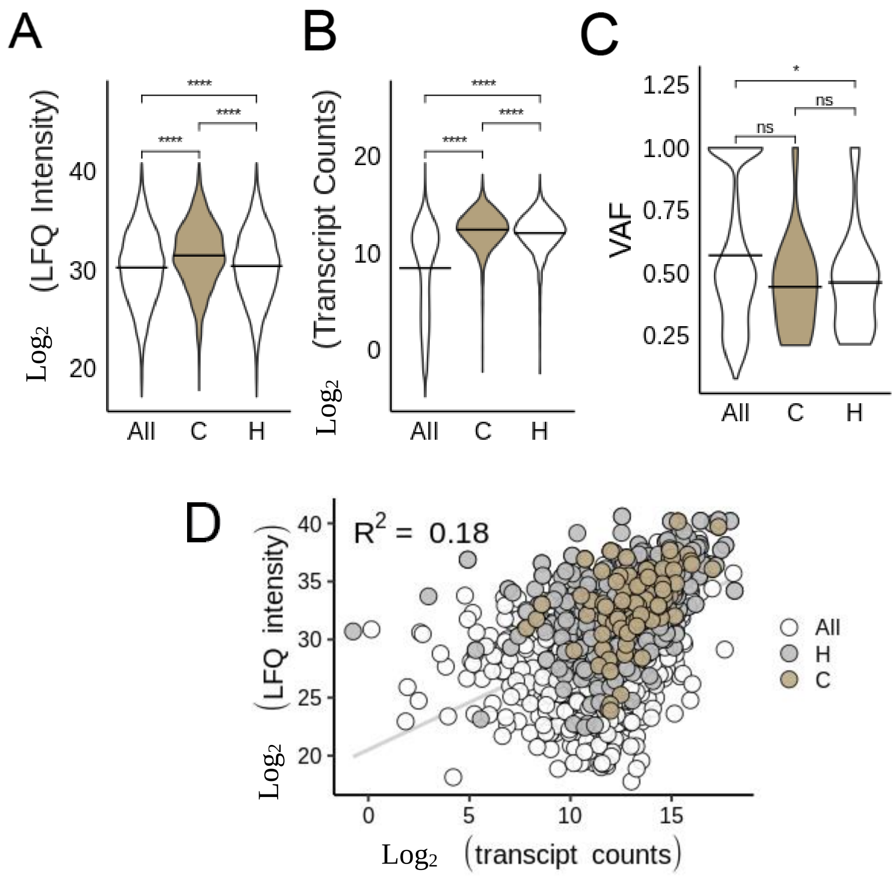

Supplementary Figure 35: A) Label free quantitation (LFQ) intensities comparisons and B) DE-seq normalized transcript comparisons from HCT-15 cell line reference database searches. C) Variant allele frequencies for gain-of-cysteines subset in HCT-15 and Molt-4 searches. D) Matched LFQ intensities and normalized transcript count correlation of variant containing proteins/transcripts; All=all proteins in LFQ search or all transcripts, C=proteins from cys-enrichment search, H=proteins from high-pH fractionation search. Statistical significance was calculated using two-sample Kolmogorov-Smirnov tests, **** $p < 0.0001$, ns $p > 0.05$. Source data are provided as a Source Data file.

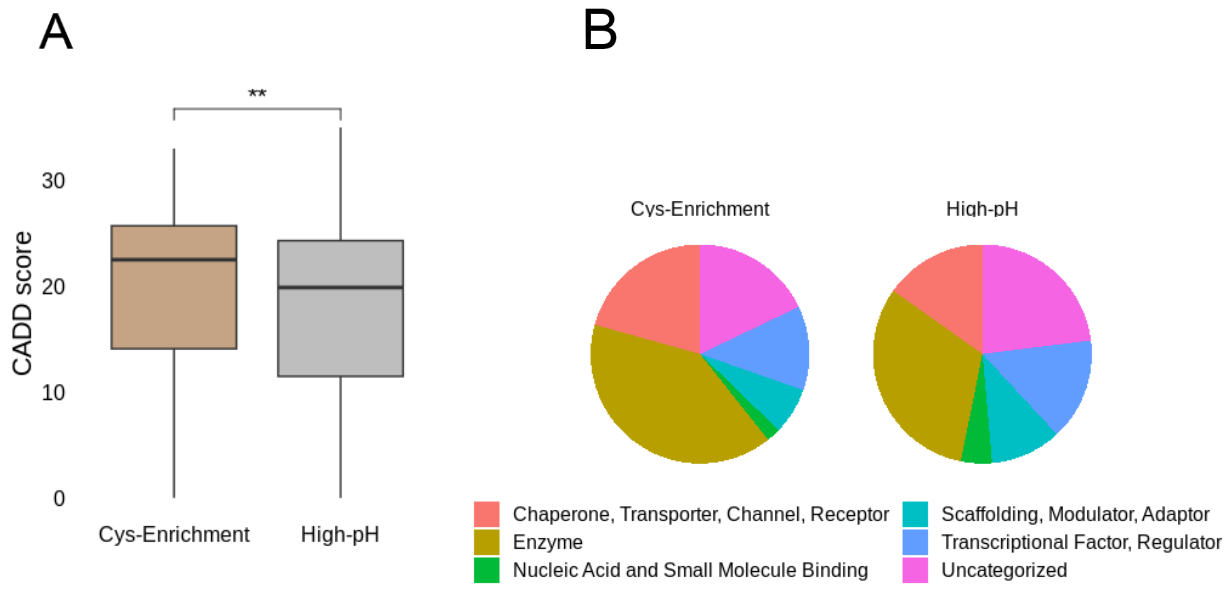

Supplementary Figure 36: A) CADD score comparison between cys-enriched and high-pH detected variants. Box plot center line, median; limits are upper and lower quartiles; 1.5x interquartile range. Statistical significance was calculated using Mann-Whitney U test, *** p < 0.01 B) Protein classes of cys-enriched and high-pH detected variants. Source data are provided as a Source Data file.

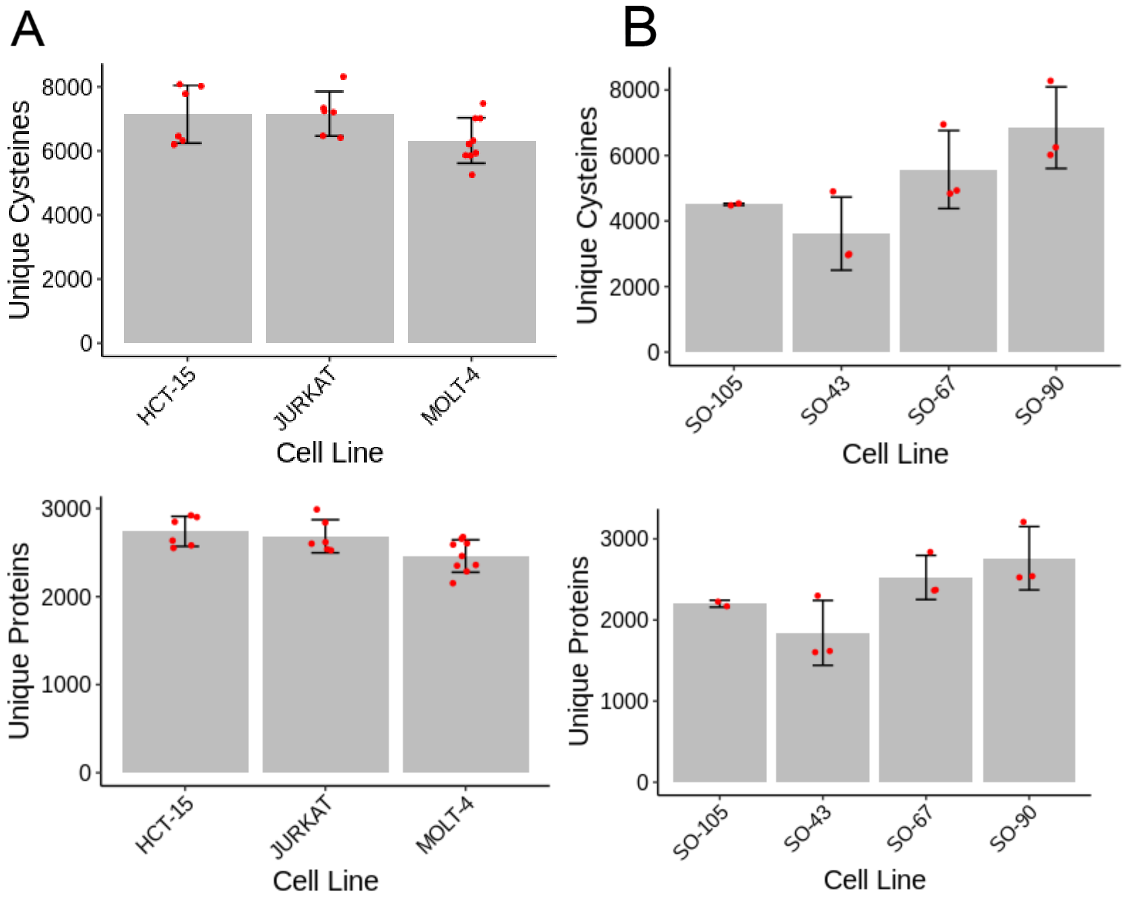

Supplementary Figure 37. Reference identifications in Figure 8 for A) KB02 datasets or B) HCT-15 datasets. Showing mean values and error bars = SD. Source data are provided as a Source Data file.

(C) Supplementary Note: Data Analysis and statistics

Fig 2B, D, S5-S9,: COSMIC Cell Lines Project Complete mutation data 'CosmicCLP_MutantExport.tsv.gz' was downloaded (release v96, 31st May 2022). For each cell line, the 'Substitution-Missense' amino acid changes were totaled up; the gained counts (example Cys, X–>C) were subtracted from the lost counts (example Cys, C–>X) to obtain net counts for each amino acid. The analysis was limited to accession numbers matching a curated set of non-redundant Ensembl transcript IDs (24,950) to limit any over/under counting due to mutations in multi-transcript proteins. **Fig S17**: ClinVar (3-28-23) data filtered for unique gene name,protein position, and amino acid change to calculate total gains and losses. **Fig 2C/3E**: common SNPs from NCBI (04-23-2018 00-common_all.vcf.gz) were filtered for missense mutations (645,395) and further filtered to include only variants in the non-redundant Ensembl transcript set. Net changes were calculated from unique 'GeneID_amino acid change identifiers'.

Fig 3B,C,D,S26: Missense table in Supplementary Data S3 (tab 22) was filtered for unique 'CellLine_Protein_AAchange' identifiers, removing transcripts IDs that result in the same amino acid changes per protein. **Fig 5B,C,D,S12-16, S19-20,S22-25:** CADD-PHRED score distributions were obtained by filtering the missense table for only single nucleotide base substitutions and uploading them as a vcf ('#CHROM', 'POS', 'ID', 'REF', 'ALT') to the CADD query tool https://cadd.gs.washington.edu/score using GRCh38-v1.6. Distribution of CADD scores shown by indicated grouping, unique 'CellLine_Protein-AAchange-CADD-score' identifiers were used to remove variants identified in both RNA and exomes. **Fig 6C, 6D-E:** Active site/binding site analysis: We consider single variant residues in or within +/- 10 amino acids in primary sequence from an annotated active/binding site range as annotated by Uniprot.

Fig 1C (and Fig S2B): Quantitative output obtained as described in *Data processing for cys-enrichment and quantitative ratio peptide analysis* for triplicate datasets. **Fig 1D**: From combined_ion_label_quant.tsv outputs for reference and variant searches, apex light - apex heavy retention times per ion were calculated for each replicate set. Mean values were obtained for ions appearing in at least two of three replicates.

Fig 4A-E, 4 (proteomics): Data was processed as described in methods for *Data processing for cys-enrichment* for duplicate datasets. **Supplementary Figure 27:** Interpro ascension numbers and interpro family/domain names were pulled for all identified variant genes using EnsDb.Hsapiens.v86 from the *ensembldb* package[8] **Fig 4E**: The provided reference FASTA was *in-silico* digested to check for cysteines theoretically detectable using the FragPipe peptide length limits of 7-50 amino acids. Cysteine peptides with loss or gain of Arg/Lys were manually checked against theoretically detectable cysteines. **Fig 6F:** SP3-Rox dataset were re-searched with 2-stage search and custom database; quantitative output obtained as described in data processing for triplicate datasets.

Fig 7: Data was processed as described in methods for *Data processing for high-pH data analysis*. **Fig 7D:** For each cell line, protein sequences from the subsetted Gencode FASTA provided in supplemental information (24,950 proteins) and as described in *custom database*

generation were used to calculate individual amino acid frequencies (# amino acid/total # amino acids) for every protein that contained a missense mutation. The total gained AA (identified in proteomic data) was used to calculate the gained frequencies (# gained AA/total missense changes identified). The fold enrichment was calculated by taking the ratio of the gained frequency to the AA frequency. **Fig 7G:** Transcript counts and LFQ data for 'High-ph' and 'All' categories were restricted to those containing at least one cysteine residue.

Fig 8: Quantitative output obtained as described in *Data processing for cys-enrichment and quantitative ratio peptide analysis.* **Fig 8C:** Ligandable cys residues were restricted to those appearing in more than one replicate, with $\log_2$ ratios > 2, and with standard deviations of ratios across replicates <= 1. **Fig 8C-G:** Values are restricted to those that appear in more than one replicate and with standard deviations =<1 for both reference and variant Cys peptides.

(D) Supplementary Note: Synthesis of Compounds

General Methods. All solution-phase reactions were performed in dried glassware under an atmosphere of dry N_2 except where water was used as a solvent. Silica gel P60 (SiliCycle) was used for column chromatography. Plates were visualized by fluorescence quenching under UV light or by staining with iodine. Other reagents were purchased from Sigma-Aldrich (St. Louis, MO), Alfa Aesar (Ward Hill, MA), EMD Millipore (Billerica, MA), Fisher Scientific (Hampton, NH), Oakwood Chemical (West Columbia, SC), Combi-blocks (San Diego, CA) and Cayman Chemical (Ann Arbor, MI) and used without further purification. ^{1}H NMR and ^{13}C NMR spectra for characterization of new compounds and monitoring reactions were collected in $CDCl_3$, CD_3OD, or DMSO-d_6 (Cambridge Isotope Laboratories, Cambridge, MA) on a Bruker AV 400 MHz spectrometer in the Department of Chemistry & Biochemistry at The University of California, Los Angeles. All chemical shifts are reported in the standard notation of parts per million using the peak of residual proton signals of the deuterated solvent as an internal reference. Coupling constant units are in Hertz (Hz). Splitting patterns are indicated as follows: br, broad; s, singlet; d, doublet; t, triplet; q, quartet; m, multiplet; dd, doublet of doublets; dt, doublet of triplets. Low-resolution mass spectroscopy was performed on an Agilent Technologies InfinityLab LC/MSD single quadrupole LC/MS (ESI source).

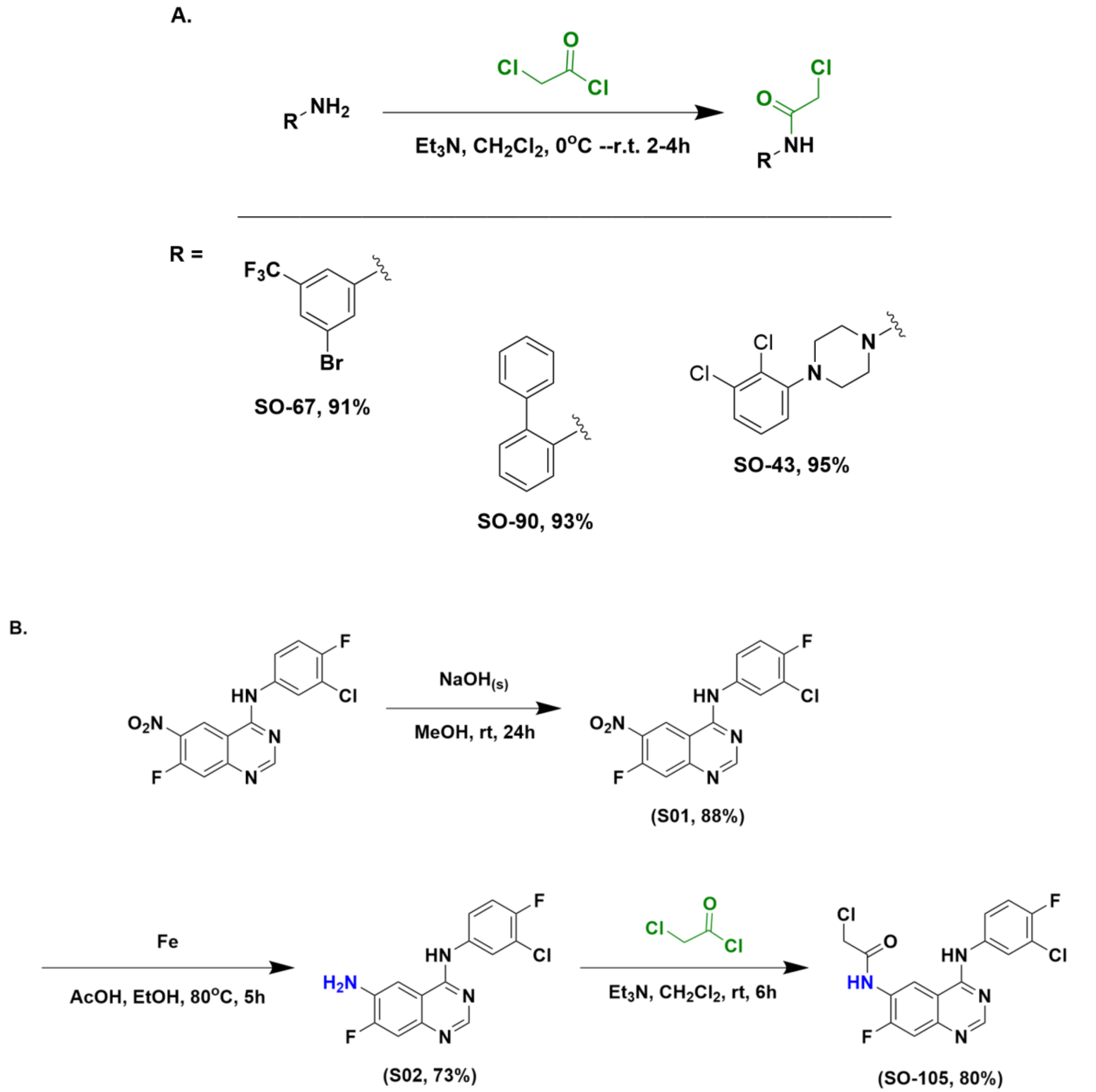

Supplementary Figure 38. Synthetic routes to obtain (A) electrophilic fragments SO-67, SO-90, and SO-43, and (B) prototype kinase inhibitor SO-105.

Synthesis

General Procedure 1:

To a solution of the amine (1 equiv.) and triethylamine (1.5 equiv.) in dichloromethane (5 mL), 2-chloroacetyl chloride (1.1 equiv.) was added dropwise and the reaction mixture was stirred at 0°C for 0.5 h then left to warm to room temperature (2 - 6h). The reaction progress was monitored via TLC. On reaction completion, the reaction mixture was poured slowly into distilled water (10ml). After separation of the phases, the organic layer was dried with anhydrous sodium sulfate, filtered

and concentrated to obtain the crude product. This crude product was then coated on silica gel, then purified via flash chromatography (in 15-30% Ethyl acetate-hexanes) to yield the analytically pure product.

Synthesis of N-(3-bromo-5-(trifluoromethyl)phenyl)-2-chloroacetamide (SO-67)

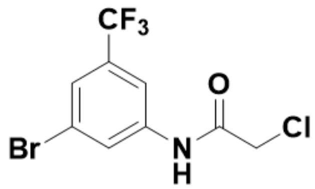

Prepared according to general procedure 1 using 3-bromo-5-(trifluoromethyl)aniline (200mg, 0.833 mmo) as the amine source. Product: white solid . **Yield**: 240 mg (91%)

^{1}H NMR (400 MHz, $CDCl_3$) δ 8.34 (s, 1H), 8.03 (s, 1H), 7.76 (s, 1H), 7.57 (s, 1H), 4.21 (s, 2H). **^{13}C NMR** (100 MHz, $CDCl_3$) δ 164.09, 138.38, 133.17, 132.84, 132.51, 132.20, 132.13, 125.93, 124.93, 123.23, 115.40, 42.73. **^{19}F NMR** (400 MHz, $CDCl_3$) δ -62.94.

HRMS (ESI-TOF) $[M+H]^+$ = $C_9H_7BrClF_3NO^+$: calculated for 315.9305 ; Found 315.9352

Synthesis of 2-chloro-1-(4-(2,3-dichlorophenyl)piperazin-1-yl)ethan-1-one (SO-43)

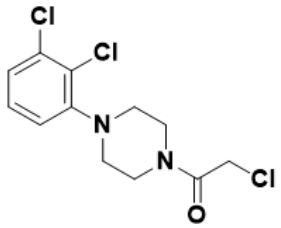

Prepared according to general procedure 1 using 1-(2,3-Dichlorophenyl)piperazine.HCl (300 mg, 1.12 mmol) as the amine source. Product: off-white solid. **Yield:** 95% (326mg).

^{1}H NMR (400 MHz, $CDCl_3$) δ 7.24 – 7.14 (m, 2H), 6.94 (dd, J = 7.8, 1.7 Hz, 1H), 4.12 (s, 2H), 3.77 (dt, J = 43.0, 4.9 Hz, 4H), 3.16 – 3.00 (m, 4H). **^{13}C NMR** (101 MHz, $CDCl_3$) δ 169.96, 165.55, 150.36, 134.26, 127.63, 125.43, 118.86, 51.47, 50.92, 46.67, 42.47, 40.76.

HRMS (ESI-TOF) $[M+H]^+$ = $C_{12}H_{14}Cl_3N_2O^+$: calculated for 307.0172 ; Found 307.0040

Synthesis of N-([1,1'-biphenyl]-2-yl)-2-chloroacetamide (SO-90)

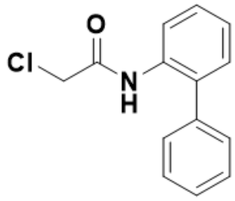

Prepared according to general procedure 1 using [1,1'-biphenyl]-2-amine (300 mg, 1.12 mmol) as the amine source. Product: off-white solid. **Yield:** 93% (410mg).

^{1}H NMR (400 MHz, $CDCl_3$) δ 8.46 (s, 1H), 8.36 (d, J = 8.2 Hz, 1H), 7.53 – 7.47 (m, 2H), 7.45 – 7.37 (m, 4H), 7.30 (dd, J = 7.6, 1.7 Hz, 1H), 7.24 (dd, J = 7.4, 1.2 Hz, 1H), 4.08 (s, 2H). **^{13}C NMR** (101 MHz, $CDCl_3$) δ 163.64 , 137.43 , 133.92 , 132.65 , 130.14 , 129.15 , 128.22 , 124.98 , 120.64 , 43.03.

HRMS (ESI-TOF) $[M+H]^+$ = $C_{14}H_{12}ClNO^+$: calculated for 246.0607 ; Found 246.0623

Synthesis of N-(3-chloro-4-fluorophenyl)-7-methoxy-6-nitroquinazolin-4-amine (S01)

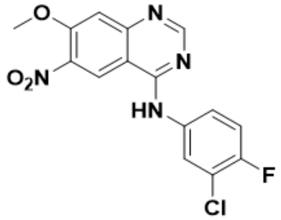

To the N-(3-chloro-4-fluorophenyl)-7-fluoro-6-nitroquinazolin-4-amine (1 g, 2.97 mmol, 1 eqv.) in methanol (23.7 mL, 551 mmol, 200 eqv.) was added sodium hydroxide pellets (1.2 g, 29.8 mmol, 10 eqv.). The reaction was refluxed overnight and monitored by TLC. Next, the reaction mixture was taken off the heat source and allowed to cool to room temperature. Then, the mixture was poured into a saturated solution of sodium bicarbonate, and then filtered under vacuum. The resultant residue was washed with water (1x), then methanol (200 mL). Then the solid residue was dried under high vacuum to yield the target compound as yellowish solid.. **Yield:** 88% (0.91g).

^{1}H NMR (400 MHz, DMSO-d$_6$) δ 9.09 (s, 1H), 8.51 (s, 1H), 8.05 (s, 1H), 7.71 – 7.64 (m, 1H), 7.43 – 7.28 (m, 2H), 4.02 (s, 3H). **^{13}C NMR** (100 MHz, DMSO-d$_6$) δ 158.12, 154.93, 154.15, 152.39, 138.63, 124.23, 123.18, 122.64, 119.22, 116.72, 109.59, 57.41.
LC-MS (ESI) [M+H]$^+$ = $C_{15}H_{11}ClFN_4O_3^+$: calculated for 348.04 ; Found 348.0

Synthesis of N^4-(3-chloro-4-fluorophenyl)-7-methoxyquinazoline-4,6-diamine (S02)

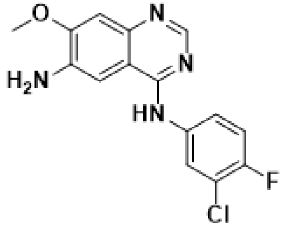

Procedure: To a stirred mixture of N-(3-chloro-4-fluorophenyl)-7-methoxy-6-nitroquinazolin-4-amine (800 mg, 0.435 mol) in ethanol (100 mL) and AcOH (5 mL) was added Fe (641 mg, 11.5 mmol) and the reaction was heated to reflux. Further, more ethanol (50 mL) and AcOH (5 mL) were added, and reflux continued for 5 h, with reaction progress monitored by LC-MS. The reaction was cooled to room temperature, when deemed complete. Next, the crude solution was filtered by passing it through celite. The filtrate was then concentrated to about one-third its original volume. The resultant precipitate was isolated and dried under high vacuum to give the target compound as a dark-brownish solid, (530mg, 73%) – confirmed by LC/MS – and this was used, as is, without further purification. **^{1}H NMR** (400 MHz, DMSO-*d*$_6$) δ 9.38 (s, 1H), 8.28 (d, J = 76.7 Hz, 2H), 7.81 (d, J = 8.9 Hz, 1H), 7.24 (d, J = 112.2 Hz, 3H), 5.37 (s, 2H), 3.96 (s, 3H). **^{13}C NMR** (101 MHz, DMSO-d$_6$) δ 153.20 , 150.73 , 137.99 , 122.92 , 106.36 , 101.30 , 56.25 .

LC-MS (ESI) [M+H]$^+$ = $C_{15}H_{13}ClFN_4O^+$: calculated for 318.01; Found 319.01

Synthesis of 2-chloro-N-(4-((3-chloro-4-fluorophenyl)amino)-7-methoxyquinazolin-6-yl) acetamide (SO-105)

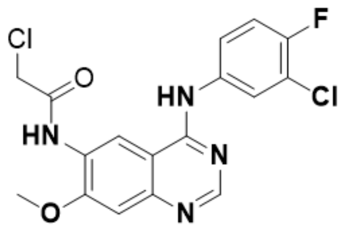

Prepared according to general procedure 1 using N^4-(3-chloro-4-fluorophenyl)-7-methoxyquinazoline-4,6-diamine (500 mg, 1.57 mmol) as the amine source. Product: pale-brownish solid. **Yield:** 80% (500mg).

^{1}H NMR (400 MHz, DMSO-*d*$_6$) δ 9.99 (s, 1H), 8.94 (s, 1H), 8.63 (s, 1H), 8.06 (dd, J = 6.8, 2.6 Hz, 1H), 7.75 (dd, J = 6.7, 2.3 Hz, 1H),

7.45 (s, 1H), 7.34 (s, 1H), 4.49 (s, 2H), 4.27 (s, 1H), 4.04 (s, 3H). **^{13}C NMR** (101 MHz, DMSO-d_6) δ 169.04, 165.68, 157.83, 155.99, 153.41, 152.99, 136.53, 127.56, 125.01, 123.77, 119.36, 116.89, 116.19, 108.83, 105.43, 56.97, 43.78.

HRMS (ESI-TOF) $[M+H]^+$ = $C_{17}H_{14}Cl_2FN_4O_2^+$: calculated for 395.0478 ; Found 395.0389

^{1}H NMR of N-(3-bromo-5-(trifluoromethyl)phenyl)-2 -chloroacetamide, **SO-67** in $CDCl_3$

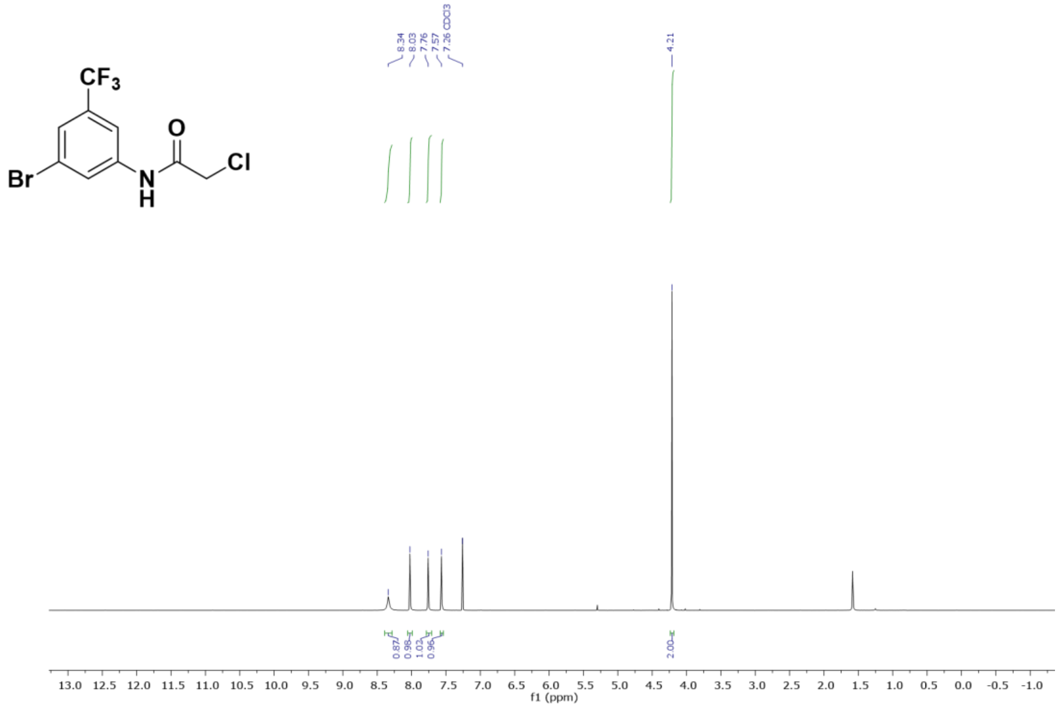

^{13}C NMR of N-(3-bromo-5-(trifluoromethyl)phenyl)-2 -chloroacetamide, **SO-67** in $CDCl_3$

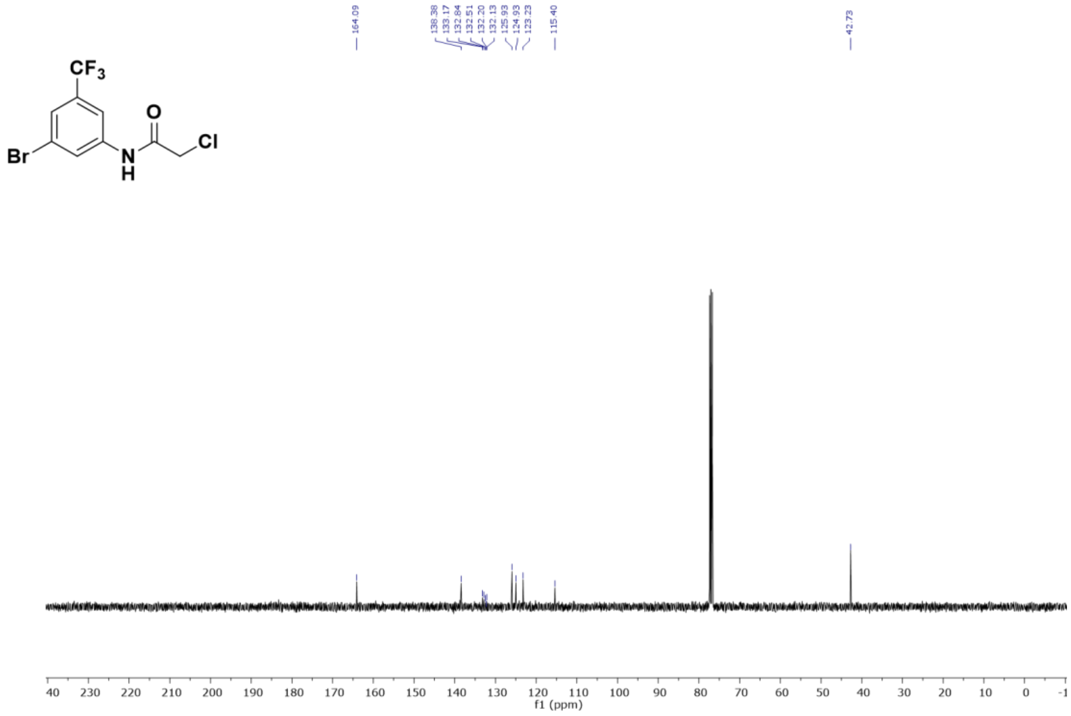

^{19}F NMR of N-(3-bromo-5-(trifluoromethyl)phenyl)-2 -chloroacetamide, **SO-67** in $CDCl_3$

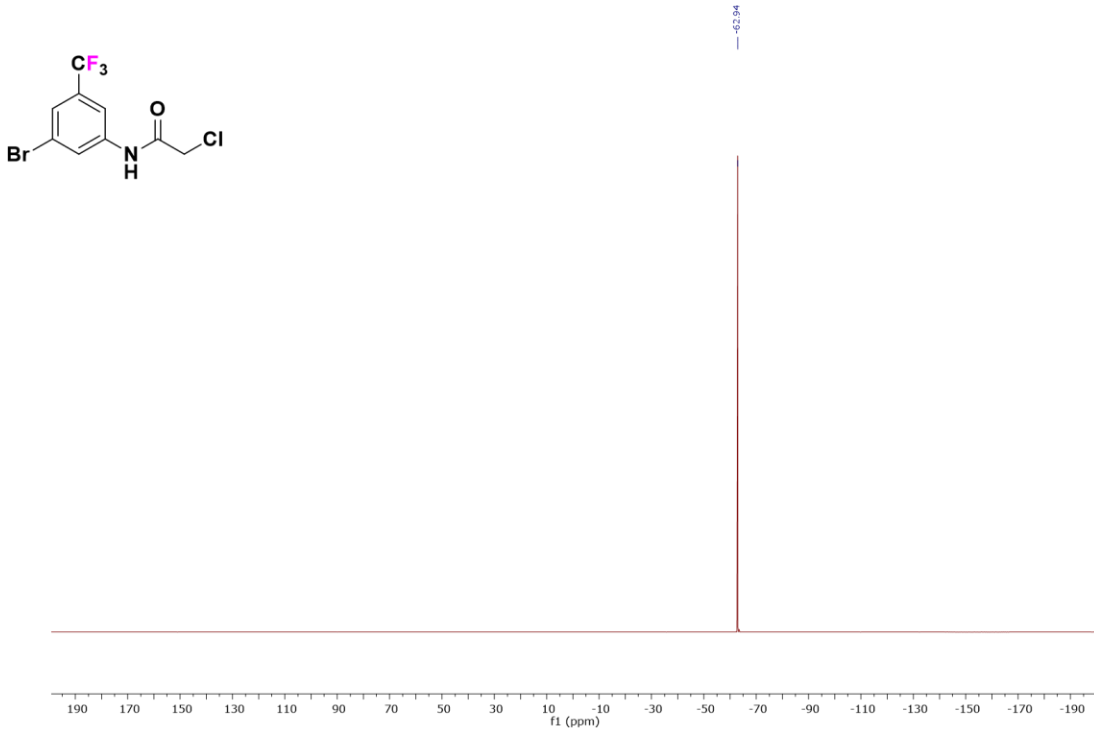

^{1}H NMR of 2-chloro-1-(4-(2,3-dichlorophenyl)piperazin-1-yl)ethan-1-one**, SO-43** in $CDCl_3$

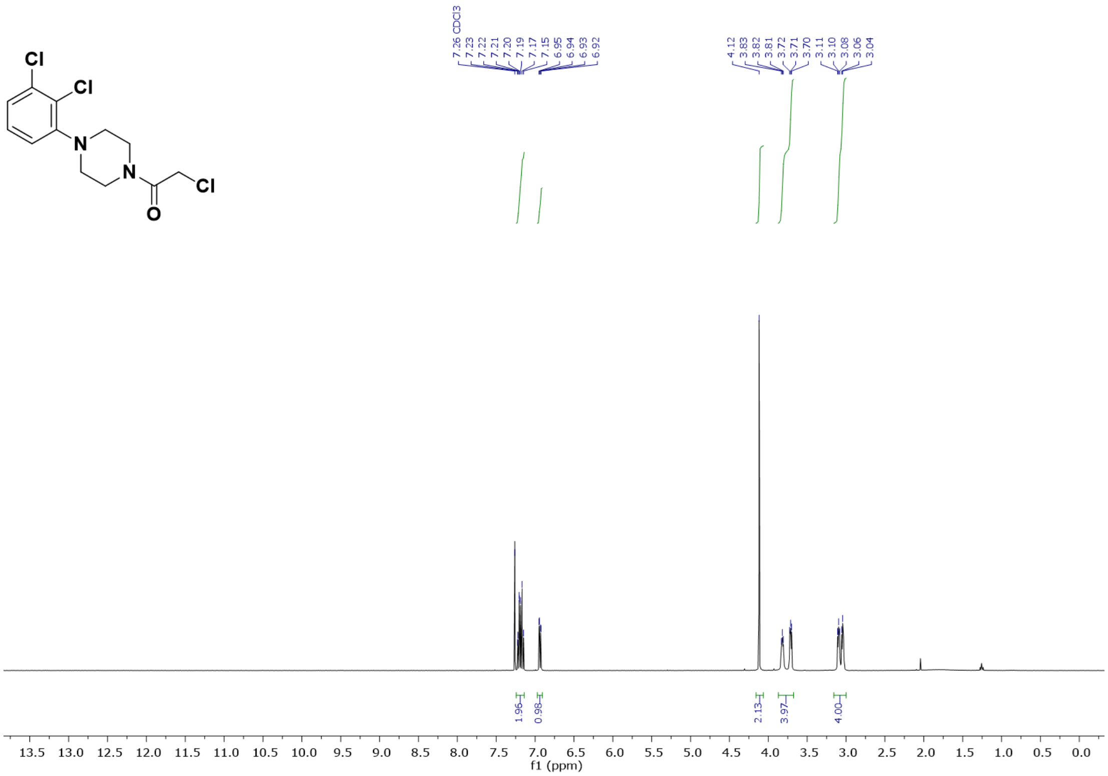

^{13}C NMR of 2-chloro-1-(4-(2,3-dichlorophenyl)piperazin-1-yl)ethan-1-one**, SO-43** in $CDCl_3$

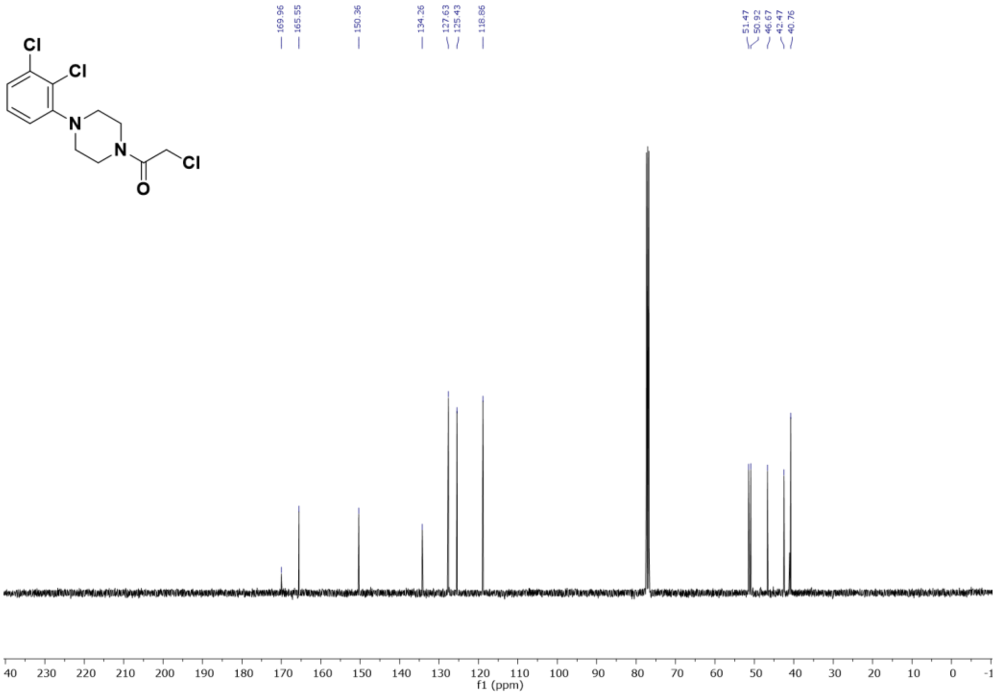

^{1}H NMR of N-([1,1'-biphenyl]-2-yl)-2-chloroacetamide , **SO-90** in $CDCl_3$

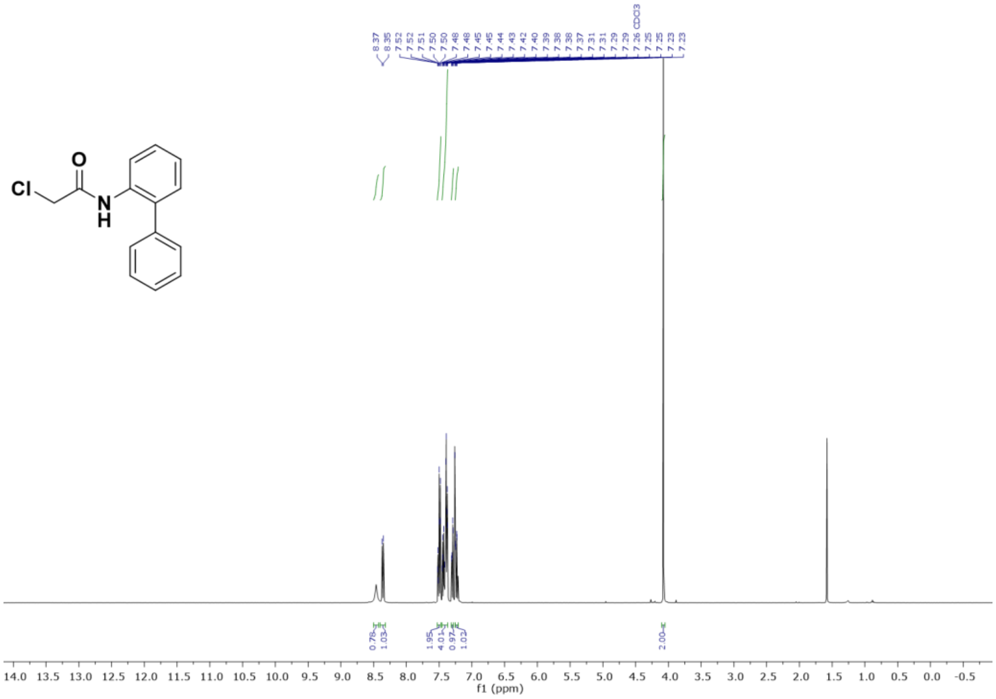

^{13}C NMR of N-([1,1'-biphenyl]-2-yl)-2-chloroacetamide , **SO-90** in $CDCl_3$

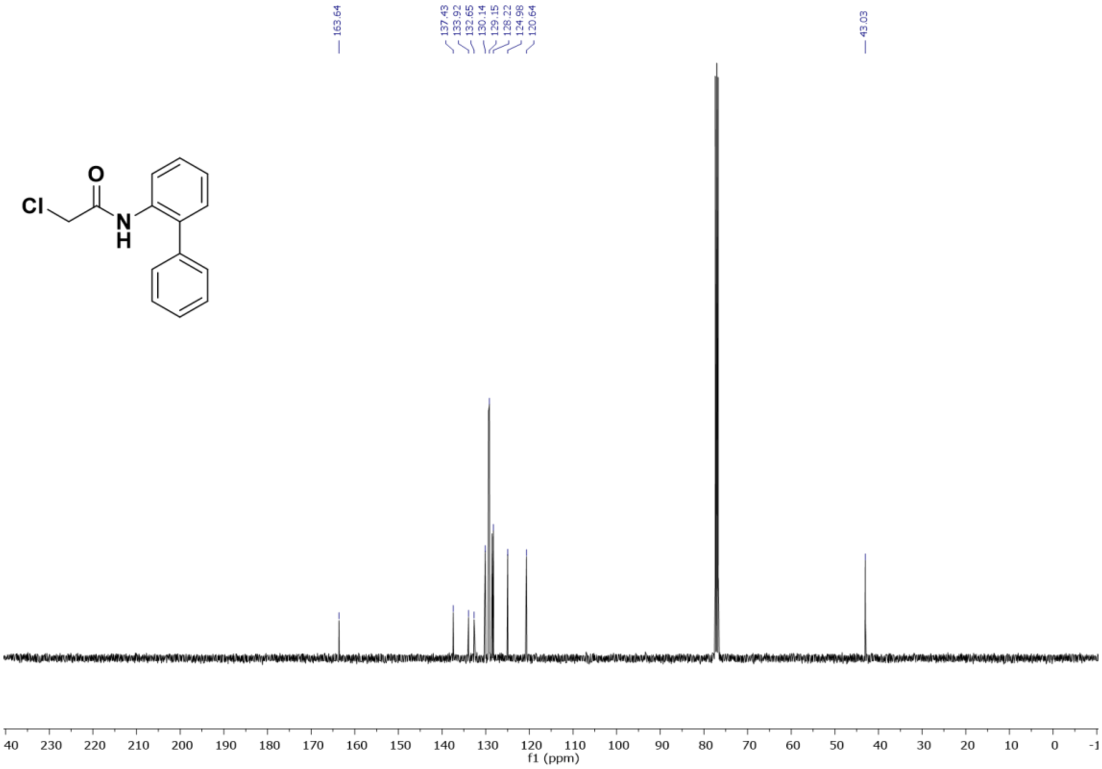

^{1}H NMR of N-(3-chloro-4-fluorophenyl)-7-methoxy-6-nitroquinazolin-4-amine **(S-01)** in $CDCl_3$

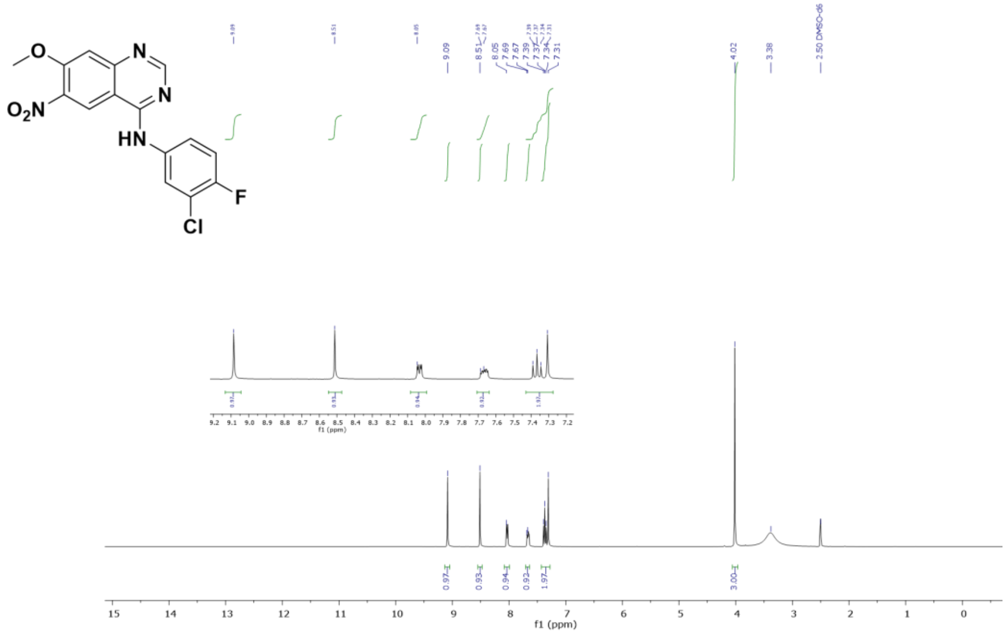

^{13}C NMR of N-(3-chloro-4-fluorophenyl)-7-methoxy- 6-nitroquinazolin-4-amine **(S-01)** in $CDCl_3$

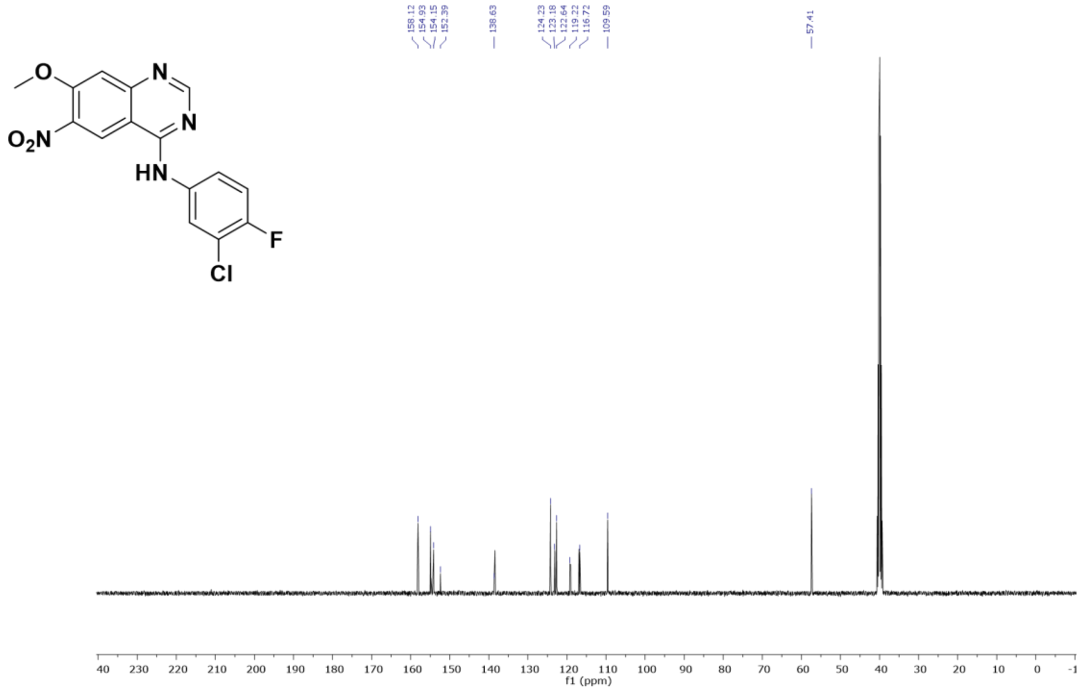

^{1}H NMR of N[4]-(3-chloro-4-fluorophenyl)-7-methoxyquinazoline- 4,6-diamine **(S-02) , in** DMSO-d_6

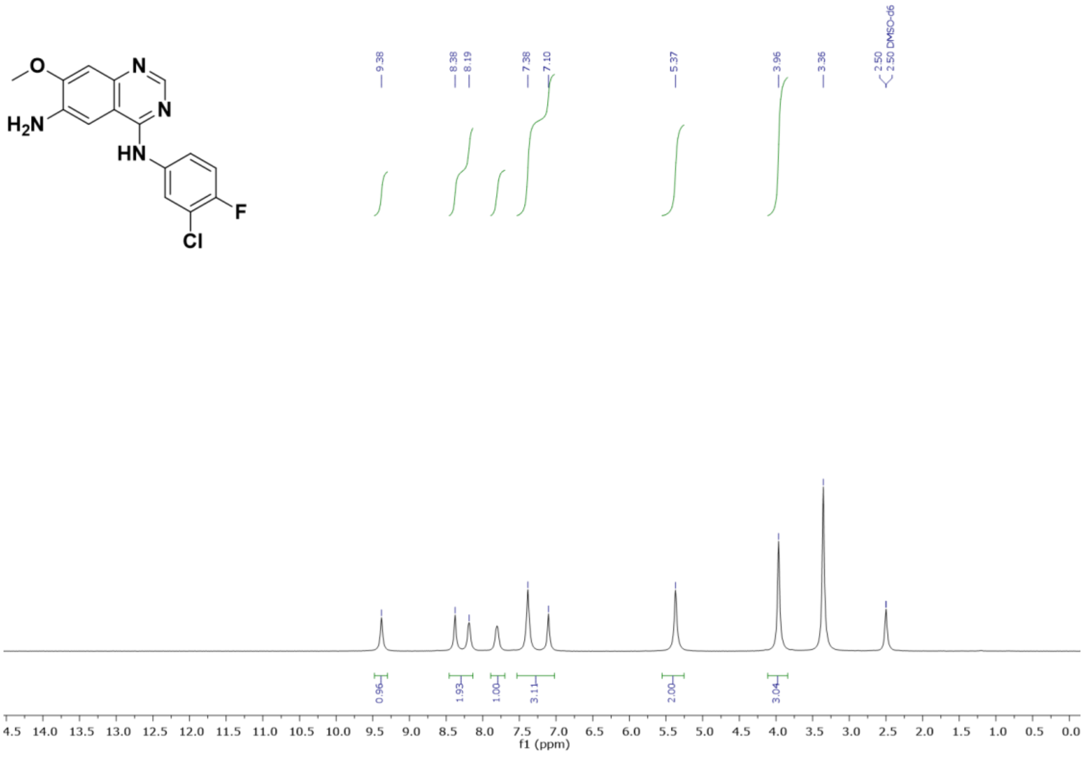

^{13}C NMR of N[4]-(3-chloro-4-fluorophenyl)-7-methoxyquinazoline-4,6-diamine **(S-02) ,** in DMSO-d_6

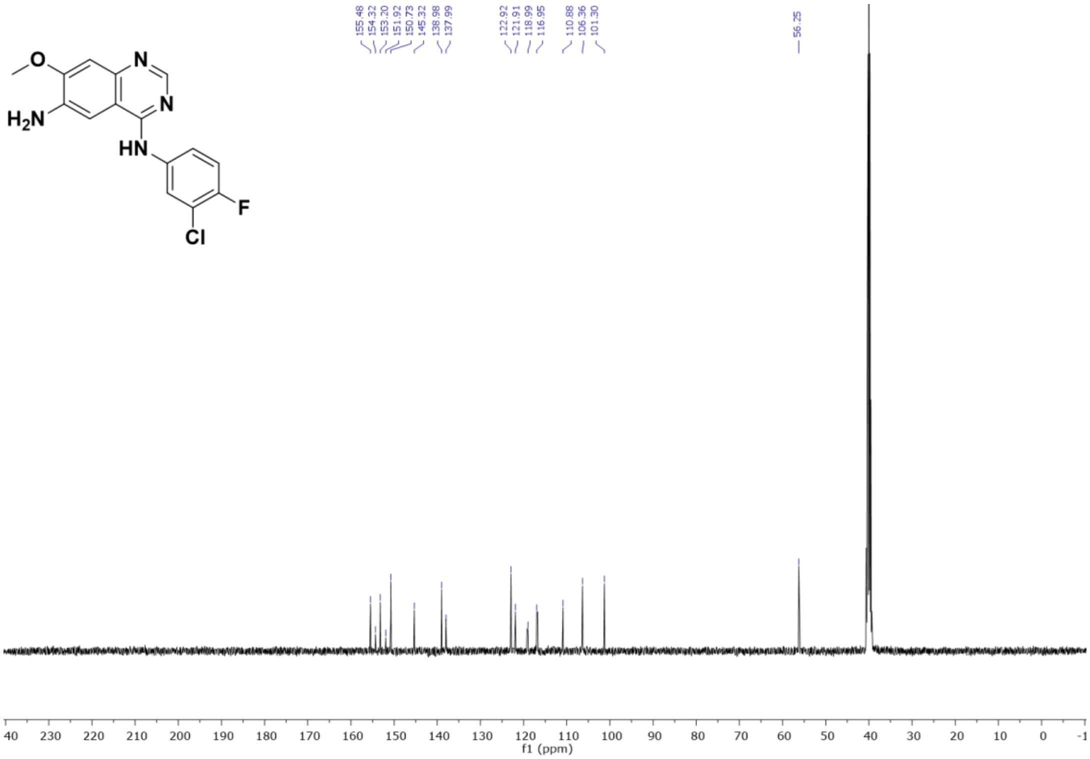

^{1}H NMR of 2-chloro-N-(4-((3-chloro-4-fluorophenyl)amino)-7-methoxyquinazolin-6-yl) acetamide, **SO-105, in** DMSO-d_6

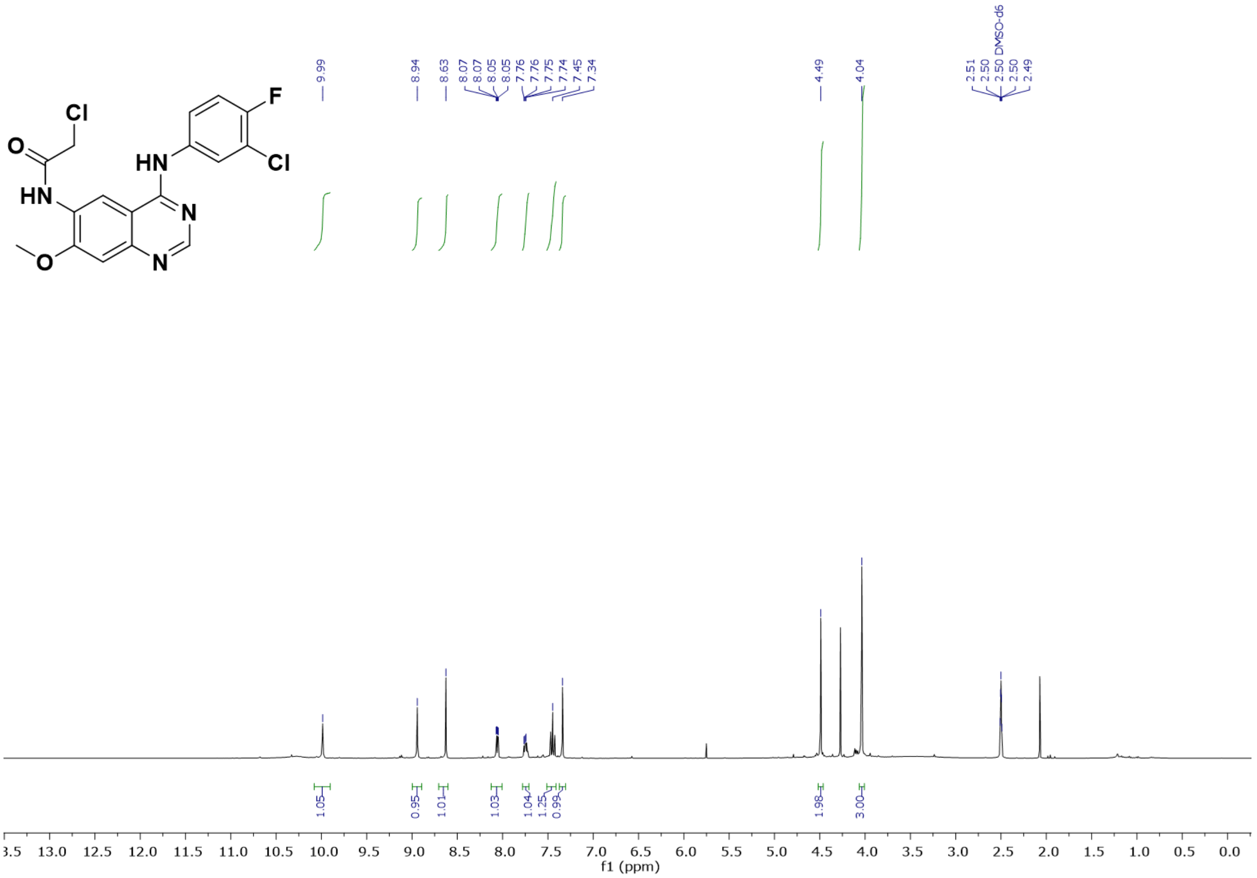

^{13}C NMR of 2-chloro-N-(4-((3-chloro-4-fluorophenyl)amino)-7-methoxyquinazolin-6-yl) acetamide, **SO-105**, in DMSO-d_6

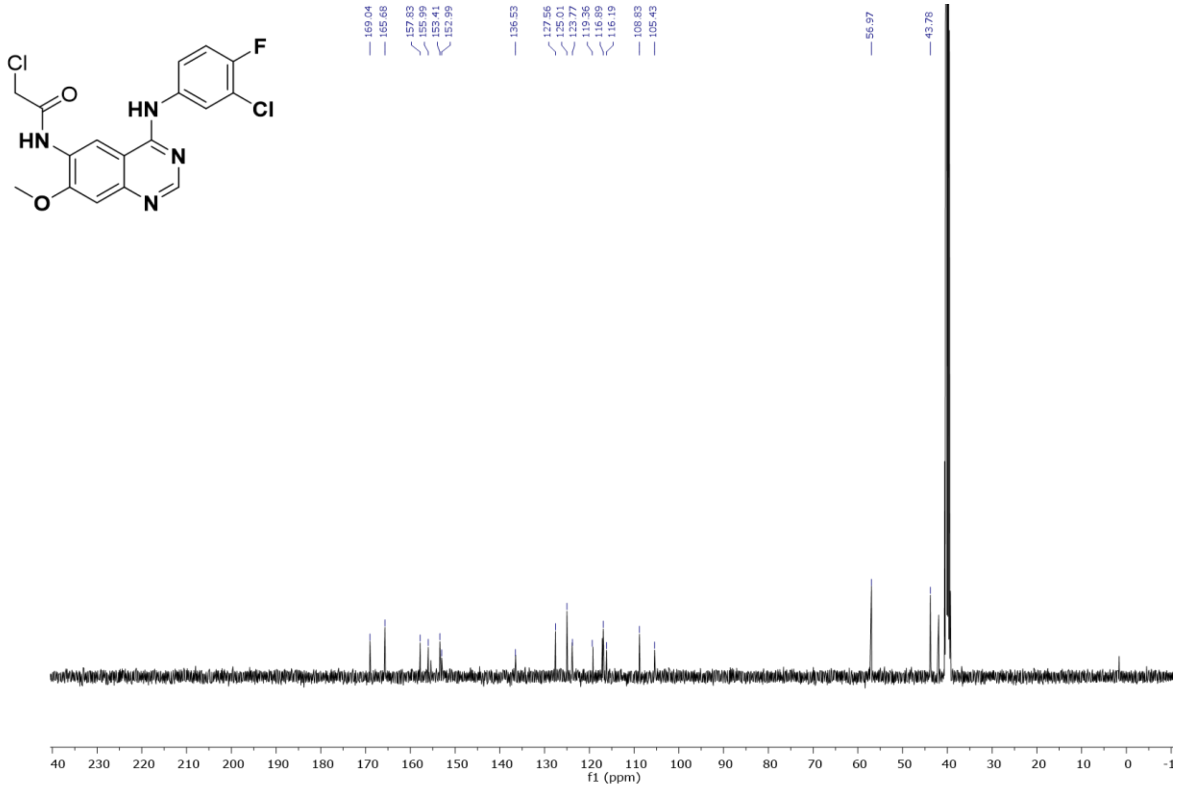

(E) Supplementary Note: Running 2-stage search with FragPipe v20+

Make sure the tools are up-to-date
FragPipe v20.0+, MSFragger version 3.8+, Philosopher version 5.0.0+

1. Set up a search as normally done with the normal reference database and with experiment specific parameters (Percolator rescoring recommended).
2. Select ‘Print decoys’ in the Validation tab
3. In the last "Run” tab, there is a checkbox called "write sub mzML":

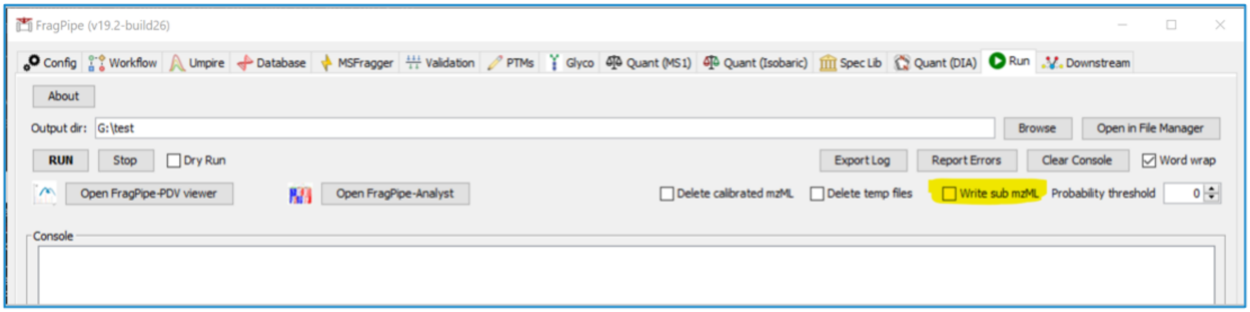

Check this box and leave the threshold set to 0.

When it is enabled, FragPipe runs the search, and writes new mzML files with "_sub.mzML" as the file name suffix, "fragpipe-second-pass.workflow", and "fragpipe-files-second-pass.fp-

manifest" files to the specified result folder. In the first-pass search, FragPipe will write decoy PSMs to psm.tsv if they pass 1% FDR.

Both "fragpipe-second-pass.workflow" and "fragpipe-files-second-pass.fp-manifest" have adjusted parameters and mzML file paths that can be directly used in the second search.

4. Name the result folder to distinguish it from the reference search ("first_pass", "reference", "1") and run the first search.
5. After the first search is finished, in your open FragPipe window, navigate to the workflow tab and load the "fragpipe-second-pass.workflow" located in your first results folder file by selecting the "custom" and clicking "load workflow". Select the fragpipe-second-pass.workflow which should then be automatically loaded.

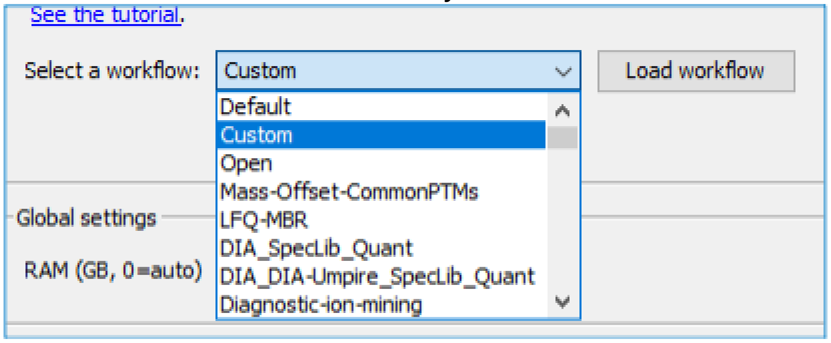

***Note**: If using Percolator: Percolator weights saving and loading is automatic. FragPipe lets Percolator save the weight files during the first-pass search. And then, in the second-pass, FragPipe gives Percolator the weight files if it can find it (the weight files are in the sub mzML files' folder)*

6. Clear all the .raw/mzML files you have loaded. Load the "fragpipe-files-second-pass.fp-manifest" by selecting load manifest and navigating to it in your first results folder. This should re-add your files with the "_sub.mzML" suffix.

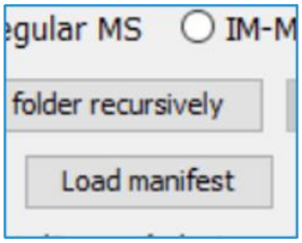

***Note**: You should not remove/add any new modifications in the second search. The MSFragger parameters should be the same, just a new database and using _sub.mzML files*

7. Go to the Database tab and navigate to the variant-containing database for your cell line.

8. Specify the reverse header (ex. "REV").

***Note**: For databases generated with https://github.com/hdesai17/chemoproteogenomics.git tool, header information containing variant info will appear as Protein/Protein-ID/Protein.Description; other database formats will depend on FragPipe compatibility. For FASTA databases lacking header information such as simple Uniprot IDs (>PXXXX), additional processing/mapping is required to obtain variant IDs.*

9. Make sure the MSFragger parameters are the same as the first search.
10. Navigate to the “Run” tab, uncheck the write sub mzML box, and specify a new results folder to distinguish variant search (“second_pass” or "variant”, “2”) and run the second search.

(F) References

1. Kong, A. T., Leprevost, F. V., Avtonomov, D. M., Mellacheruvu, D. & Nesvizhskii, A. I. MSFragger: Ultrafast and comprehensive peptide identification in mass spectrometry-based proteomics. *Nat. Methods* **14**, 513–520 (2017).
2. Teo, G. C., Polasky, D. A., Yu, F. & Nesvizhskii, A. I. Fast Deisotoping Algorithm and Its Implementation in the MSFragger Search Engine. *J. Proteome Res.* **20**, 498–505 (2020).
3. Yang, K. L. *et al.* MSBooster: Improving Peptide Identification Rates using Deep Learning-Based Features. 2022.10.19.512904 Preprint at https://doi.org/10.1101/2022.10.19.512904 (2022).
4. Käll, L., Canterbury, J. D., Weston, J., Noble, W. S. & MacCoss, M. J. Semi-supervised learning for peptide identification from shotgun proteomics datasets. *Nat. Methods* **4**, 923–925 (2007).
5. Nesvizhskii, A. I., Keller, A., Kolker, E. & Aebersold, R. A Statistical Model for Identifying Proteins by Tandem Mass Spectrometry. *Anal. Chem.* **75**, 4646–4658 (2003).
6. Obenchain, V. *et al.* VariantAnnotation : a Bioconductor package for exploration and annotation of genetic variants. *Bioinformatics* **30**, 2076–2078 (2014).
7. Julio, A. R. *et al.* Pervasive aggregation and depletion of host and viral proteins in response to cysteine-reactive electrophilic compounds. *bioRxiv* 2023.10.30.564067 (2023) doi:10.1101/2023.10.30.564067.
8. Rainer, J., Gatto, L. & Weichenberger, C. X. ensembldb: an R package to create and use Ensembl-based annotation resources. *Bioinformatics* **35**, 3151–3153 (2019).
