# Supplementary material for: Chemoproteogenomic stratification of the missense variant cysteinome
Source: Nat Commun. 2024 Oct 28;15:9284. doi: 10.1038/s41467-024-53520-x (PMC11519605; doi:10.1038/s41467-024-53520-x)
Supplement: Supplementary file 3 — Description of Additional Supplementary Files [file 41467_2024_53520_MOESM3_ESM.pdf]

Description of Additional Supplementary Files

File Name: Supplementary Data 1
Description: Heavy/light biotin and TMT validation with command line and GUI implementation of 2-stage searches. Datasets related to figure 1 and supplemental figures 2-4

File Name: Supplementary Data 2
Description: Analyses of COSMIC Cell Lines Project, ClinVar, and dbSNP. Datasets related to figure 2, 3E, and supplemental figures 5-9, 15, and 17.

File Name: Supplementary Data 3
Description: Missense variant annotations for cell lines derived from RNA-seq and exome-seq data. Datasets related to figures 3, 5, 6C,6 E and supplemental figures 1,10-14,16,19-20, 22-27.

File Name: Supplementary Data 4
Description: Proteomics data from cysteine enrichment datasets and HLA experiments. Datasets related to figure 4, 6 and supplemental figures 1,18, 21, 27-29.

File Name: Supplementary Data 5
Description: Proteomics data from high-pH datasets. Datasets related to figure 7 and supplemental figures 30-36.

File Name: Supplementary Data 6
Description: Proteomics data from ligandability analysis. Datasets related to figure 8 and supplemental figure 37.

File Name: Supplementary Data 7
Description: List of raw mass spectrometry files, description, cell line, and associated figure.

File Name: Supplementary Data 8
Description: Construct /plasmid sequences and primers used in the study.
