# Supplementary material for: Chemoproteogenomic stratification of the missense variant cysteinome
Source: Nat Commun. 2024 Oct 28;15:9284. doi: 10.1038/s41467-024-53520-x (PMC11519605; doi:10.1038/s41467-024-53520-x)
Supplement: Supplementary file 12 — Reporting Summary [file 41467_2024_53520_MOESM12_ESM.pdf]

nature portfolio

Corresponding author(s): Backus

Last updated by author(s): Sep 14, 2024

Reporting Summary

Nature Portfolio wishes to improve the reproducibility of the work that we publish. This form provides structure for consistency and transparency in reporting. For further information on Nature Portfolio policies, see our Editorial Policies and the Editorial Policy Checklist.

Statistics

For all statistical analyses, confirm that the following items are present in the figure legend, table legend, main text, or Methods section.

n/a	Confirmed	
☐	☒	The exact sample size (*n*) for each experimental group/condition, given as a discrete number and unit of measurement
☐	☒	A statement on whether measurements were taken from distinct samples or whether the same sample was measured repeatedly
☐	☒	The statistical test(s) used AND whether they are one- or two-sided<br>*Only common tests should be described solely by name; describe more complex techniques in the Methods section.*
☒	☐	A description of all covariates tested
☐	☒	A description of any assumptions or corrections, such as tests of normality and adjustment for multiple comparisons
☐	☒	A full description of the statistical parameters including central tendency (e.g. means) or other basic estimates (e.g. regression coefficient) AND variation (e.g. standard deviation) or associated estimates of uncertainty (e.g. confidence intervals)
☐	☒	For null hypothesis testing, the test statistic (e.g. *F*, *t*, *r*) with confidence intervals, effect sizes, degrees of freedom and *P* value noted<br>*Give P values as exact values whenever suitable.*
☒	☐	For Bayesian analysis, information on the choice of priors and Markov chain Monte Carlo settings
☒	☐	For hierarchical and complex designs, identification of the appropriate level for tests and full reporting of outcomes
☒	☐	Estimates of effect sizes (e.g. Cohen's *d*, Pearson's *r*), indicating how they were calculated

Our web collection on statistics for biologists contains articles on many of the points above.

Software and code

Policy information about availability of computer code

Data collection	LC-MS/MS proteomic data were collected with an Easy-nLC 1200 attached to an Orbitrap Tribrid Eclipse mass spectrometer using the Xcalibur (v4.6.67.17) software.<br>Immunoblots and fluorescent gels were imaged using a ChemiDoc™ MP Imaging System(Bio-Rad #12003153) running Image Lab Touch v2.2.0.08.
Data analysis	Raw data collected by LC-MS/MS were searched with MSFragger (v3.6 and v3.7) and FragPipe (v19.0 and 19.1). Statistics and visualization in R studio version 2023.01.01+494 and R version 4.3.1 and GraphPad Prism v9.4.1

For manuscripts utilizing custom algorithms or software that are central to the research but not yet described in published literature, software must be made available to editors and reviewers. We strongly encourage code deposition in a community repository (e.g. GitHub). See the Nature Portfolio guidelines for submitting code & software for further information.

Data

Policy information about availability of data

All manuscripts must include a data availability statement. This statement should provide the following information, where applicable:

- Accession codes, unique identifiers, or web links for publicly available datasets
- A description of any restrictions on data availability
- For clinical datasets or third party data, please ensure that the statement adheres to our policy

Mass spectrometry data files are available in the PRIDE repository: PXD040696, PXD040737
Sequencing data is deposited in Sequence Read Archive (SRA) as BioProject PRJNA997729
Publicly available databases used:
COSMIC v96 (https://cancer.sanger.ac.uk/cell_lines)
dbSNP database (4-23-18) (https://www.ncbi.nlm.nih.gov/snp/)
ClinVar (09-03-22) (https://www.ncbi.nlm.nih.gov/clinvar/)
The UniProt Consortium (https://www.uniprot.org/)
DisProt (https://disprot.org/)
Phosphosite (https://www.phosphosite.org/homeAction.action)
Protein Data Bank (https://www.rcsb.org/)

Human research participants

Policy information about studies involving human research participants and Sex and Gender in Research.

Reporting on sex and gender	N/A
Population characteristics	N/A
Recruitment	N/A
Ethics oversight	N/A

Note that full information on the approval of the study protocol must also be provided in the manuscript.

Field-specific reporting

Please select the one below that is the best fit for your research. If you are not sure, read the appropriate sections before making your selection.

☒ Life sciences ☐ Behavioural & social sciences ☐ Ecological, evolutionary & environmental sciences

For a reference copy of the document with all sections, see nature.com/documents/nr-reporting-summary-flat.pdf

Life sciences study design

All studies must disclose on these points even when the disclosure is negative.

Sample size	All proteomic experiments were performed in with at least 2 biological replicates.
Data exclusions	No data were excluded from the analyses.
Replication	Replicates were used as indicated in figure legends, method section, and text.
Randomization	Plates of cells were randomized to control/treatment groups.
Blinding	Blinding of the researcher was not relevant as data were collected with constant acquisition parameter.

Reporting for specific materials, systems and methods

We require information from authors about some types of materials, experimental systems and methods used in many studies. Here, indicate whether each material, system or method listed is relevant to your study. If you are not sure if a list item applies to your research, read the appropriate section before selecting a response.

Materials & experimental systems

n/a	Involved in the study
☐	☒ Antibodies
☐	☒ Eukaryotic cell lines
☒	☐ Palaeontology and archaeology
☒	☐ Animals and other organisms
☒	☐ Clinical data
☒	☐ Dual use research of concern

Methods

n/a	Involved in the study
☒	☐ ChIP-seq
☒	☐ Flow cytometry
☒	☐ MRI-based neuroimaging

Antibodies

Antibodies used	anti-FLAG rabbit antibody (14793, Cell Signaling)<br>IRDye® 800CW Goat anti-Rabbit IgG (102673-330, VWR)<br>IRDye® 680RD Goat anti-Rabbit IgG (102673-410, VWR)<br>GAPDH rabbit antibody (2118S, Cell Signaling)<br>CUL1 Rb Antibody (4995, Cell Signaling)<br>anti HA-Tag Rb mAb (C29F4, Cell Signaling)<br>anti DDDDK-Tag Rb mAb (D6W58, Cell Signaling)<br>IRDye 800CW Goat anti-Rabbit IgG (LI-COR)β�Actin Rb mAb (AC038, ABclonal)
Validation	All antibodies were purchased from vendors that validate their antibodies using siRNA-mediated protein knockdown and/or overexpression.

Eukaryotic cell lines

Policy information about cell lines and Sex and Gender in Research

Cell line source(s)	HEC-1B(HTB-113), HCT-15(CCL-225), MOLT-4(CRL-1582), MEWO(HTB-65), CACO-2(HTB-37), NCI-H661(HTB-183), NCI-H358(CRL-5807), NCI-H1437(CRL-5872), NCI-H2122(CRL-5985), JURKAT-A3(CRL-2570), MIA-PACA-2(CRM-CRL-1420)<br>all sourced from ATCC (https://www.atcc.org/)
Authentication	Authenticated by chromosomal analysis and short tandem repeat profiling. We also ensure that the features of the cells match the ATCC descriptions (i.e. morphological features, splitting frequency).
Mycoplasma contamination	All cell lines are tested for Mycoplasma contamination monthly
Commonly misidentified lines<br>(See ICLAC register)	No commonly misidentified cell lines were used in this study.
